# Predicting T790M mutation status in non-small cell lung cancer based on radiomics: A systematic review and meta-analysis

**DOI:** 10.1371/journal.pone.0353257

**Published:** 2026-07-08

**Authors:** Hongyang Chen, Bingjie Fan, Mengqi Yuan, Dandan Wang, Chenxi Qiao, Na Qiu, Xiaomin Quan, Wei Hou

**Affiliations:** 1 Department of Oncology, Guang’anmen Hospital, China Academy of Chinese Medical Sciences, Beijing, China; 2 Department of Graduate School, Beijing University of Chinese Medicine, Beijing, China; 3 Capital Medical University, Beijing, China; 4 Beijing Hospital of Traditional Chinese Medicine, Beijing, China; 5 China Academy of Chinese Medical Sciences, Beijing, China; 6 Faculty of Chinese Medicine and State Key Laboratory of Mechanism and Quality of Chinese Medicine, Macao University of Science and Technology, Macao, Macao SAR, China; SUNY Upstate Medical University Hospital, UNITED STATES OF AMERICA

## Abstract

**Background:**

Epidermal growth factor receptor tyrosine kinase inhibitors (EGFR-TKIs) have revolutionized the prognosis for patients with EGFR-mutant lung cancer. The emergence of the T790M resistance mutation compromises the efficacy of EGFR-TKI therapy. Therefore, assessing EGFR T790M mutation status during non-small cell lung cancer (NSCLC) treatment is crucial for improving NSCLC prognosis.

**Method:**

PubMed, Embase, Web of Science databases, China National Knowledge Infrastructure, and Wanfang as primary sources were systematically searched up to January 1, 2026. To assess the risk of bias and study quality, we employed the Quality Assessment of Diagnostic Accuracy Studies (QUADAS) tool and the Radiomics Quality Score version 2.0 (RQS). The diagnostic accuracy of radiomics for detecting T790M in NSCLC patients was evaluated by calculating the area under the curve (AUC), sensitivity, specificity, and accuracy for each study.

**Results:**

This meta-analysis analyzed 13 studies with 2,654 patients. The pooled AUC, sensitivity, and specificity of internal validation models were 0.91, 0.73, and 0.95, respectively. The pooled AUC, sensitivity, and specificity of external validation models were 0.81, 0.73, and 0.87, respectively. Subgroup analysis revealed that imaging examinations derived from lung and mediastinal metastases achieved the highest sensitivity (0.76; 95% CI, 0.73–0.79), whereas those based on brain metastases exhibited the highest specificity (0.95; 95% CI, 0.95–0.96). The high specificity of the lung/mediastinal models was further confirmed in external validation (0.96; 95% CI, 0.95–0.98). Compared with CT, MRI-based models demonstrated a trade-off in internal validation: lower sensitivity (0.72 vs. 0.75) but significantly higher specificity (0.96 vs. 0.80). Notably, in external validation, CT achieved superior sensitivity (0.96, 95% CI 0.94–0.99). ITK-SNAP demonstrated higher sensitivity (internal: 0.76 [95% CI, 0.73–0.79]; external: 0.76 [95% CI, 0.67–0.84]) and lower specificity (internal: 0.80 [95% CI, 0.76–0.85]; external: 0.83 [95% CI, 0.70–0.95]). When stratified by a median RQS exceeding 20, higher-scoring studies were associated with higher pooled sensitivity (0.76 [95% CI, 0.70–0.82]) but a lower specificity (0.85 [95% CI, 0.79–0.90]). While in external validation, RQS ≤ 20 demonstrated higher sensitivity (0.75 [95% CI, 0.68–0.82], *P* < 0.001). Integrating clinical factors with radiomics improved sensitivity but reduced specificity compared with radiomics-only models (0.79 vs. 0.72 and 0.81 vs. 0.95, respectively). A similar sensitivity-specificity trade-off was observed with standardized data processing (sensitivity: 0.76 vs. 0.72; specificity: 0.80 vs 0.95).

**Conclusion:**

Radiomics, as a non-invasive detection method, has demonstrated significant potential in predicting the T790M mutation status in NSCLC, showing promising clinical application prospects based on retrospective evidence. However, further standardization and validation are required in future studies.

**Systematic review registration:**

https://www.crd.york.ac.uk/PROSPERO/view/CRD420251130164 (CRD420251130164).

## Introduction

Lung cancer remains the leading cause of cancer-related deaths globally, with non-small cell lung cancer (NSCLC) accounting for 85% of cases [1,2]. Recently, epidermal growth factor receptor tyrosine kinase inhibitors (EGFR-TKIs) have transformed outcomes for patients with EGFR-mutated lung cancer [3]. In the National Comprehensive Cancer Network (NCCN) guidelines, many first- or second-generation EGFR-TKIs, such as afatinib, gefitinib, and erlotinib, are recommended for first-line treatment [4]. However, patients treated with EGFR-TKIs eventually develop acquired resistance after approximately 9–14 months of therapy, which may lead to a worsening prognosis [5]. Notably, T790M is the primary cause of acquired resistance, with 60% of patients developing T790M after initial response to first-line EGFR-TKI therapy [6,7]. Osimertinib has demonstrated efficacy in patients with T790M-positive patients who have progressed from reversible EGFR-TKI treatments and is also effective in patients with primary T790M mutation [8,9]. Although osimertinib has been approved as a first-line treatment for patients with advanced EGFR mutation-positive NSCLC, due to its high cost, some patients still prioritize lower-cost first or second-generation EGFR-TKIs. Additionally, for adjuvant therapy after early-stage lung cancer surgery, osimertinib, icotinib (first-generation), and afatinib (second-generation) have all received corresponding indications. Drug selection requires comprehensive consideration of factors including the patient’s specific stage, mutation type, and physical condition. Therefore, evaluating EGFR T790M mutation status during the course of NSCLC and early identifying T790M resistance mutations, especially in patients with disease progression, can facilitate timely adjustment of targeted treatment strategies, which is crucial for improving the prognosis of NSCLC.

In clinical practice, assessment of T790M mutation status relies on plasma circulating tumor DNA (ctDNA) detection and biopsy [10]. While tissue biopsy remains definitive, its invasiveness, patient discomfort, and risk of complications—including potential promotion of metastasis—limit its utility [11]. Moreover, it captures only a limited spatial profile of the disease and is susceptible to intratumoral heterogeneity [12]. Liquid biopsy via ctDNA has emerged as a promising alternative; however, its sensitivity remains constrained by low tumor DNA fraction in plasma and dilution by normal cell-free DNA [13]. Both approaches are costly, provide only a static genomic snapshot, and are typically employed post-treatment, thereby offering no guidance for initial therapeutic strategy. Consequently, there is an urgent unmet need for predictive tools that are cost-effective, minimally invasive, and longitudinally applicable to assess T790M status.

Radiomics is an emerging field that employs mathematical analysis and computer-aided detection to extract features from medical images. As a non-invasive technique, radiomics can derive quantitative characteristics from diverse imaging modalities, providing detailed descriptions of tumor heterogeneity, imaging features, and tumor-related risk factors such as size and malignancy [14,15]. Consequently, it enhances the accuracy of disease diagnosis, treatment planning, and monitoring [16,17]. Furthermore, the role of artificial intelligence and radiomics in building imaging biobanks is of critical importance, as such repositories are essential for enabling personalized care and advancing precision medicine [18]. Recent studies indicate that machine learning (ML) models constructed using features extracted from metastatic lesions via multi-sequence MRI can distinguish patients with T790M-resistant NSCLC [19]. *Li* et al. developed a nomogram using radiomic scores from non-contrast and contrast-enhanced CT, achieving an AUC of 0.853 in predicting T790M resistance within 14 months, highlighting its potential for early detection [20]. However, the lack of standardized radiomics workflows limits the robustness and reproducibility of these models.

Although radiomics-based predictive models for T790M gene expression levels show promise in NSCLC, they remain immature and may be constrained by methodological limitations, inter-study variability, and issues of generalizability and reproducibility. Only through systematic evaluation can they be incorporated into clinical practice. This study aims to systematically review and comprehensively summarize the application of radiomics in the early identification of T790M gene mutations in NSCLC, with a focus on diagnostic performance, sensitivity, and specificity, providing potential reference tools for clinicians to assess T790M status and improve the accuracy of early diagnosis.

## Materials and methods

### Study protocol and registration

The current study was prepared in accordance with the Preferred Reporting Items for Systematic Reviews and Meta-Analyses (PRISMA) guidelines [21], ensuring a structured and transparent methodology. The review protocol was registered and approved in the International Prospective Register of Systematic Reviews (PROSPERO) database (Registration ID: CRD420251130164).

### Literature search strategy

According to the PRISMA statement, two authors (HC and BF) independently conducted a comprehensive database search using PubMed, Embase, Web of Science databases, China National Knowledge Infrastructure, and Wanfang as primary sources, covering the time period from each database’s inception to the publication of studies up to January 1, 2026. The search employed a combination of Medical Subject Headings (MeSH) and keywords associated with radiomics, NSCLC, T790M, and prediction. Target literature was additionally obtained by reviewing the references of included studies. The specific search strategy is detailed in **Table S2**.

### Inclusion criteria and screening

In this study, we established inclusion and exclusion criteria based on the PICOS questions. The inclusion criteria were as follows: (1) Population (P): participants with NSCLC; (2) Intervention (I): intervention involving imaging examination in all participants; (3) Comparator (C): negative T790M gene test result; (4) Outcome (O): diagnostic results (T790M + /-) presented in a 2 × 2 test performance table; (5) Study design (S): ML studies evaluating the diagnostic value of imaging for NSCLC, published in peer-reviewed journals.

The exclusion criteria were as follows: (1) Insufficient outcome information for data analysis; (2) Conference papers, case reports, systematic reviews, etc.; (3) Unfinished studies or published research including unfinished studies, reviews, conference papers, abstracts, and case reports; (4) Duplicate reports; (5) Studies without full-text availability.

### Study selection and data extraction

Two authors (HC and BF) recorded data in standardized spreadsheets. Any discrepancies were resolved through consultation with a third author (WH). Extracted data included: (1) Study characteristics: first author, publication year, country, data source, study design; (2) Patient characteristics: sample size, training/validation set distribution, tumor stage; (3) Radiomics-related parameters (imaging modality, tumor lesion segmentation method, region of interest (ROI) size, feature extraction software, feature type); (4) Validation methods; (5) Model performance metrics: evaluation indicators for predictive models, including AUC values, sensitivity, specificity, along with their 95% confidence intervals (95%CI)s, and true positives (TP), false positives (FP), true negatives (TN), and false negatives (FN).

If sensitivity and specificity were not directly reported, we reconstructed the 2 × 2 contingency table by extracting true-positive and false-positive rates from available receiver operating characteristic (ROC) curves using GetData Graph Digitizer 2.24 software [21]. To mitigate selection bias, AUC values were derived from all validation set data within prediction models based on radiomics features, with stratified reporting for internal and external validation [22].

### Quality assessment

The included studies were assessed using the Radiomics Quality Score version 2.0 (RQS) checklist and QUADAS-2 [23–25]. Two authors (XQ and CQ) conducted independent assessments, resolving any discrepancies through consultation with the third author (DW). All radiomics methodologies employed the latest version of Radiomics Quality Score (RQS 2.0, accessed November 23, 2025, at https://www.radiomics.world/rqs2), a framework proposed by *Lambin* and colleagues in 2017 [26] to evaluate the quality of radiomics research reports. RQS 2.0 evaluates radiomics studies across 42 assessment dimensions within 9 key domains, rewarding or penalizing them to promote optimal scientific practice. The maximum achievable score is 56 points (100%) [25]. The QUADAS-2 tool is the standard for diagnostic accuracy meta-analyses, assessing bias and applicability in patient selection, indicator detection, reference standards, and study procedures. Responses were recorded as “yes,” “no,” or “unclear” in RevMan 5.4.

### Data analysis

This meta-analysis employed STATA (version 14) and RevMan (5.4) for statistical analysis. A bivariate random-effects model was used to pool data across different validation datasets. We calculated sensitivity, specificity, positive likelihood ratio (PLR), and negative likelihood ratio (NLR) along with their corresponding 95% confidence intervals (CIs). Additionally, we constructed summary receiver operating characteristic (SROC) curves and calculated the AUC using a random-effects model to assess the diagnostic performance of the pooled studies [27]. Based on AUC, a rough classification of accuracy is as follows: 0.90−1 (excellent), 0.80–0.90 (good), 0.70–0.80 (fair), 0.60–0.70 (poor), and 0.50–0.60 (very poor).

Heterogeneity was assessed using *I*^*2*^ and Q statistics, with *I*^*2*^ values categorized as low, moderate, or high (0–50%, 50–75%, and >75%). A random-effects model was used in all analyses to account for expected between-study heterogeneity. Forest plots displayed sensitivity and specificity across studies, with pooled estimates calculated.

Exploratory meta-regression analyses and subgroup analyses evaluated multiple covariates to identify sources of heterogeneity, including clinical characteristics, model calibration methods, study design, data source, imaging modality, tumor lesion segmentation method, ROI size, feature extraction software, model combination characteristics, reference standard, and model validation methods.

To assess the impact of individual studies on the overall estimate, sensitivity analysis employed a univariate diagnostic odds ratio (DOR) model to identify potential outliers affecting the pooled results. This was conducted by sequentially excluding one study at a time from the meta-analysis calculations. Any identified outliers were reanalyzed to validate the robustness of the results. Deeks’ funnel plot asymmetry tests assessed publication bias [28]. Statistical significance was defined as *P* < 0.05. A random-effects model was used to evaluate study pooling and effect size, accommodating the distribution characteristics of true effects in heterogeneous studies.

## Results

### Screening and selection of articles

**Fig 1:**
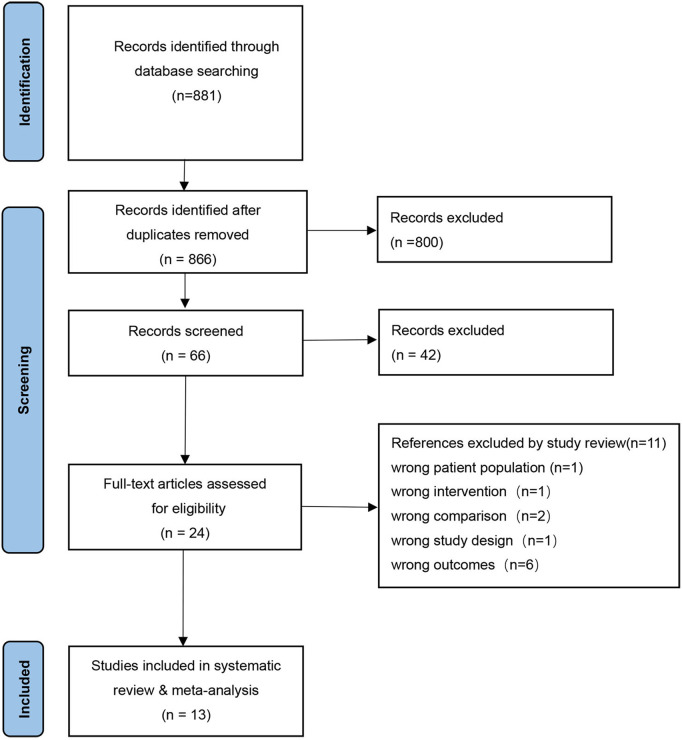
Flowchart demonstrating the process of selecting studies.

A systematic literature search was conducted according to the predetermined strategy, identifying a total of 881 publications. After removing 15 duplicate records, 866 publications underwent title and abstract screening, resulting in the exclusion of 842 irrelevant studies. The full texts of the remaining 24 papers were assessed for eligibility. After comprehensive review, 11 articles were excluded for inconsistency with the study objectives. This selection process ultimately yielded 13 eligible articles [19,20,29–39] that conformed to the PICOS criteria for inclusion in the final meta-analysis. The screening process and PRISMA flow diagram are shown in Fig 1.

### Study and patient characteristics

These 13 articles were published between 2022 and 2025, involving a total of 2,654 patients (Tables 1, 2 and S4). All included studies originated from China. One study [36], involving 5 datasets, utilized multicenter data sources, while twelve studies [19,20,29–35,37–39], comprising 160 datasets, employed single-center data sources.

**Table 1:** General characteristics information of Studies Included in the Systematic Review.

			Training cohort			Internal validation cohorts			External validation cohorts					Imaging equipment
study	Country	No.	T790M +/-	Age, (year)	M/F	T790M +/-	Age, (year)	M/F	T790M +/-	Age, (year)	M/F	Populations TNM stage	Reference Standard	
Fan	China	100	24	58.17 ± 7.13	8/16	12	59.00 ± 10.56	4/8	8	63.25 ± 5.70	3/5	NSCLC with spinal metastasis	blood samples	MRI
2022			30	60.40 ± 9.76	15/15	16	60.94 ± 11.52	8/8	10	62.90 ± 11.46	4/6	IV		
Fan1	China	160	21	58.57 ± 9.60	9/12	11	55.45 ± 7.75	3/8	13	58.62 ± 9.61	3/10	NSCLC with BM IV	blood samples	MRI
2023			32	57.72 ± 8.77	10/12	16	59.13 ± 8.64	5/11	17	55.24 ± 8.57	7/10			
Fan2	China	110	21	59.95 ± 9.13	7/14	11	52.82 ± 6.91	5/6	13	58.62 ± 9.61	4/9	NSCLC with BM and IV	blood samples	MRI
2023			32	58.38 ± 8.52	11/21	16	54.75 ± 8.14	4/12	17	55.24 ± 8.57	7/10			
Li1	China	162	55	NA	NA	NA	NA	NA	28	NA	NA	metastatic NSCLC IV	genetic test reports	NECT+CECT
2023			58	NA	NA	NA	NA	NA	21	NA	NA			
Li2	China	233	68	59.13 ± 7.78	28/40	NA	NA	NA	27	61.15 ± 7.88	4/23	NSCLC with BM IV	pathological biopsy or blood samples	MRI
2023			108	59.13 ± 7.78	47/61	NA	NA	NA	30	61.91 ± 5.79	7/23			
Lv	China	405	194	57.54 ± 9.24	31/64	NA	NA	NA	115	57.14 ± 8.91	23/32	NSCLC with BM IV	pathological biopsy or blood samples	MRI
2023	(lesion-level)		426	56.03 ± 10.30	75/124	NA	NA	NA	120	56.31 ± 10.21	20/36			
Tang	China	346	86	NA	NA	NA	NA	NA	89	NA	NA	advanced NSCLC IV	pathological biopsy or blood samples	NECT+CECT
2023			125	NA	NA	NA	NA	NA	46	NA	NA			
Cui	China	80	21	56.10 ± 7.59	7/14	11	60.18 ± 11.14	5/6	NA	NA	NA	NSCLC with BM IV	genetic test reports	MRI
2024			32	56.59 ± 8.27	11/21	16	58.31 ± 9.07	4/12	NA	NA	NA			
Lu	China	274	90	65.30 ± 9.90	52/38	38	65.26 ± 10.81	26/12	NA	NA	NA	NSCLC III/IV	pathological biopsy	CT
2024			102	64.16 ± 10.72	43/59	44	66.11 ± 10.98	19/25	NA	NA	NA			
Wu	China	125	18	60.70 ± 10.80	7/11	NA	NA	NA	NA	NA	NA	NSCLC with BM IV	genetic test reports	MRI
2024			107	65.20 ± 11.30	43/64	NA	NA	NA	NA	NA	NA			
Xiong	China	120	45	65.70 ± 10.60	20/25	19	65.40 ± 10.60	14/5	NA	NA	NA	NSCLC IIIB-IV	pathological biopsy	CT
2024			39	65.40 ± 10.60	15/24	17	68.20 ± 9.00	4/13	NA	NA	NA			
Xiong	China	116	42	65.50 ± 10.40	22/20	18	65.10 ± 10.50	11/7	NA	NA	NA	NSCLC IIIB-IV	pathological biopsy	CT
2025			39	65.10 ± 10.50	15/24	17	70.00 ± 9.40	4/13	NA	NA	NA			
Zhang	China and the USA	423	19	NA	NA	0	NA	NA	9	NA	NA	NSCLC NA	genetic test reports	CT
2025			166	NA	NA	NA	NA	NA	129	NA	NA			

Abbreviations: BM, brain metastases; F, female; M, male; NA, not available; No., number of patients; NSCLC, non-small cell lung cancer.

**Table 2:** Radiomics-related information of Studies Included in the Systematic Review.

Author Year	Imaging loaction	Segmentation method	AI method	Combined clinical parameters	The best model	Classification model	Feature Extraction Software	Feature selection method	Segmentation Software	Data source	Region of Interest	Standardization
Fan 2022	Spine	manual	ML	Yes	nomogram models incorporating radiomics and smoking	LR	Pyradiomics	LASSO and 10-fold cross-validation	ITK-SNAP	multi-center	ROI 2D and 3D	No
Fan1 2023	BTI and VPE	manual	ML	No	radiomics signature-combined with VPE.	LR	Pyradiomics	LASSO and 10-fold cross-validation	ITK-SNAP	multi-center	ROI 2D and 3D	Yes
Fan2 2023	Brain, POA, and TAA	manual	ML	No	radiomics signature-combined with POA and TAA	LR	Pyradiomics	LASSO and 10-fold cross-validation	ITK-SNAP	single center	ROI 3D	Yes
Li1 2023	Lung and mediastinum	manual	ML	No	nomogram combined the rad-score calculated by NECT and CECT model	LR	Pyradiomics	LASSO,10-fold cross-validation, and MinMaxScaler	3D slicer	single center	ROI NA	Yes
Li2 2023	Brain metastases	manual	ML	No	DWI model	RF	Pyradiomics	MIC,10-fold cross-validation, Boruta, and SMOTE	3D Slicer	multi-center	ROI 2D and 3D	Yes
Lv 2023	Brain metastases	manual	ML	No	lesion-level model consisting of rad-scores	RF	Pyradiomics	LASSO,10-fold cross-validation, MinMaxScaler, MIC, and SMOTE	3D Slicer	multi-center	ROI NA	Yes
Tang 2023	Lung and mediastinum	manual	ML	Yes	nomogram combined the rad-score calculated by NECT and CECT model	Artificial Neural Network, Adaptive Boosting, Fast Nearest Neighbor, XGB, DT, NB, SVM, RF and LR	Pyradiomics	LASSO and 5-fold cross-validation, Boruta, Minimum Redundancy Maximum Relevance, the Relief, InfGain, GainRatio, Gini, and DistEuclid	3D Slicer	single center	VOI 3D	Yes
Cui 2024	Brain metastases	manual	ML	Yes	T1C and T2W MRI and clinical feature fusion	LR	Pyradiomics	LASSO and mRMR	ITK-SNAP	single center	ROI 3D	No
Lu 2024	Lung and mediastinum	manual	ML	Yes	nomogram combined CT images and clinical features	DT, KN; LR, NB, RF, SVM, and XGB	Pyradiomics	LASSO and 5-fold cross-validation	ITK-SNAP	single center	ROI 3D	Yes
Wu 2024	Brain metastases	manual	ML	No	SVM-SMOTE oversampling method in combination with the XGBoost classifie	LR, SVM, RF, and XGB	Pyradiomics	LASSO and 10-fold cross-validation	1.5T Signa™ HDxt scanner	single center	ROI 2D	No
Xiong 2024	Lung and mediastinum	manual	ML	Yes	SVM-radiomics model-clinical model	LR, RF, and SVM	GE AnalysisKit	LASSO and 3,5- cross-validations	ITKSNAP	single center	VOI 3D	No
Xiong 2025	Lung and mediastinum	manual	ML	Yes	Combined use of Radscore and clinical characteristics	LR	GE AnalysisKit	LASSO,10-fold cross-validation	ITK-SNAP	single center	ROI 3D	Yes
Zhang 2025	Lung and mediastinum	manual	ML	Yes	Radiomics-Clinical	MLP, SVM, RF, LR, KNN, NB, and LDA	Pyradiomics	LASSO and 5-fold cross-validation	3D Slicer	multi-center	ROI 2D and 3D	Yes

Note: LR, logistic regression; LASSO, Least Absolute Shrinkage and Selection Operator; DT, decision tree; KNN, knearest neighbors; NB, naïve Bayes; RF, random forest; SVM, support vector machines; XGBoost, XGB, and extreme gradient boosting; TAA, tumor active area; POA, peritumoral edema area; VPE, volume of peritumoral edema; BTI, brain-to-tumor interface, ML, machine learning; ROI, region of interest; LDA, linear discriminant analysis.

Within the radiomics workflow, six studies [19,29–31,34,38], involving 127 datasets, used device MRI to image brain metastases from NSCLC, while the remaining studies [31–33,35,36,39], with 30 datasets, utilized CT to image lung and mediastinal regions; only one study [37] used MRI to image spinal metastasis. *Fan* et al. [29,30] performed imaging of tumor active areas (TAA) and peritumoral edema (POA) in two separate studies. For tumor lesion segmentation, manual segmentation was commonly employed to delineate regions of interest within tumors.

Five studies [19,20,31,32,36], comprising 17 datasets, utilized 3D Slicer software for sampling, seven studies [29,30,33,35,37–39], involving 44 datasets, used ITK-SNAP software for sampling. Eleven studies utilized the open-source software PyRadiomics for feature extraction and representative texture features.

LASSO was the most common method for feature selection [19,29–39]. Classification methods mainly employed included LR [29–39], SVM [32–36], LDA [36], RF [19,20,32–36], XGB [32–34], DT [32,33], and NB [32,33,36]. Nine studies [19,20,29–33,36,39], comprising 41 datasets, standardized extracted imaging feature values during data processing. All studies employed ML algorithms for model building and validation. To enhance model robustness, seven studies [19,20,29–31,34,37,39] used 10-fold cross-validation, three studies [32,33,36] used 5-fold cross-validation, and one study [35] used both 3-fold and 5-fold cross-validation.

Additionally, six studies [33,35–39], involving 22 datasets, combined clinical factors with radiomics features for model construction. Seven studies [19,20,29–31,36,37], involving 25 datasets, employed external validation, while nine studies [29,30,32–35,37–39], with 140 datasets, used internal validation.

### Quality assessment

**Fig 2:**
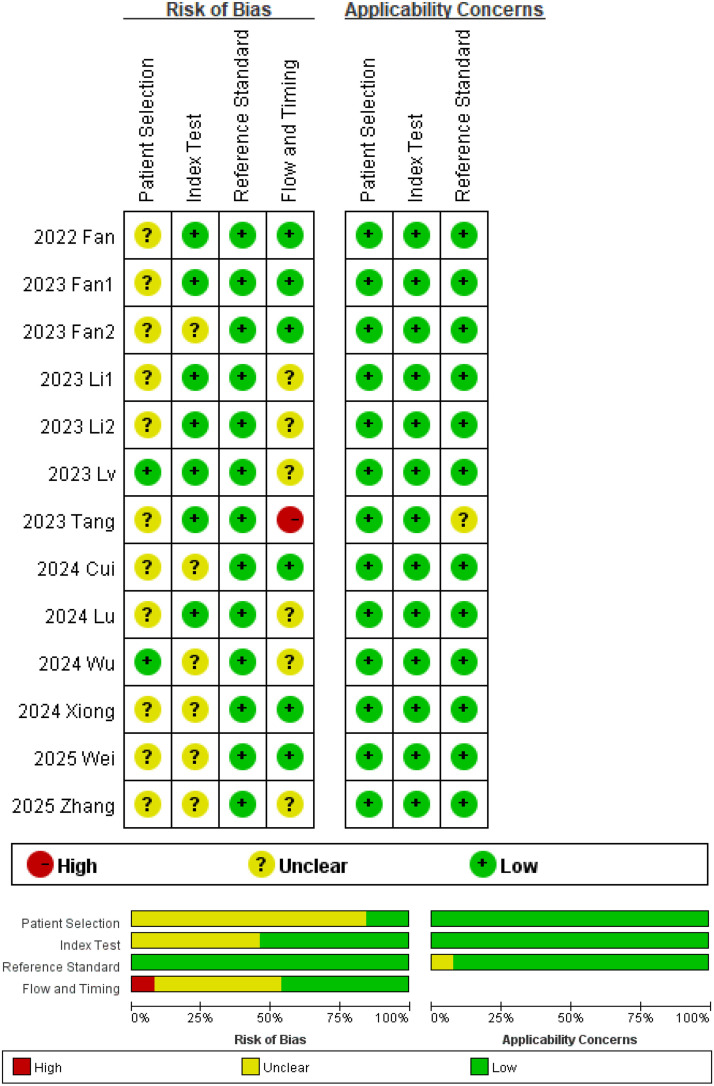
The summary of the quality assessment of the included studies following QUADAS-2.

We assessed the quality of the selected studies using the QUADAS-2 tool, as shown in Fig 2. Overall, the quality of all studies was acceptable. In the patient selection domain, the risk of bias was unclear for 11 studies because continuity was not mentioned during patient selection. In the index test domain, seven studies demonstrated low risk of bias, and all studies explicitly specified the reference standard domain. By flow and timing domain, six studies reported insufficient information regarding the interval between performing radiomics analysis and the reference standard; one study was identified as having a higher risk of bias, primarily because patients in two cohorts received two different gold standards. Regarding clinical applicability assessment, only one study had an unclear risk of bias concerning the applicability evaluation of the gold standard due to differing gold standards; the other studies showed a low risk of bias.

We used the RQS 2.0 checklist shown in Table S3 to assesse the quality of all radiomics studies. Among the 9 domains, the median overall RQS was 20 (range 18–25), with an overall quality score of 36.26%. All studies were retrospective. Furthermore, no studies scored in the areas of prospective validity, applicability and sustainability, or clinical deployment.

### Diagnostic test accuracy analysis

**Fig 3:**
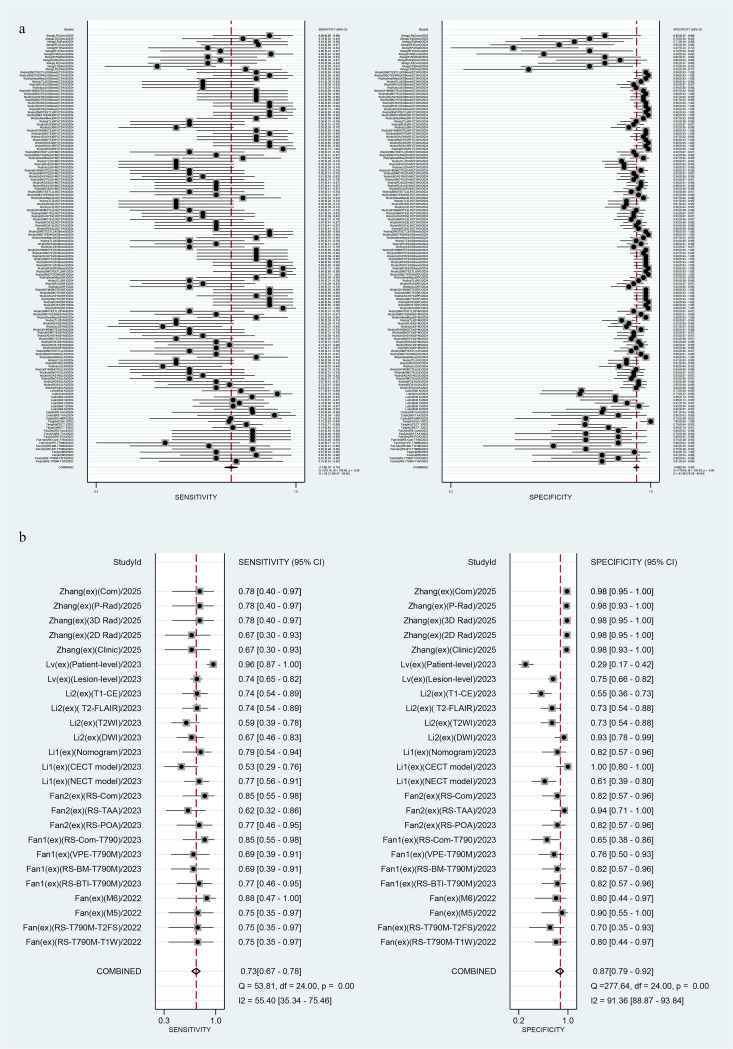
Forest plots of sensitivity and specificity with corresponding 95% CIs of the radiomics model in predicting T790M mutation status for non-small cell lung cancer on (a) internal validation and (b) external validation. The diamond at the bottom of each panel represents the pooled estimate. The size of each square is proportional to the study’s weight in the random-effects meta-analysis. The dashed vertical line indicates the pooled summary estimate.

**Fig 4:**
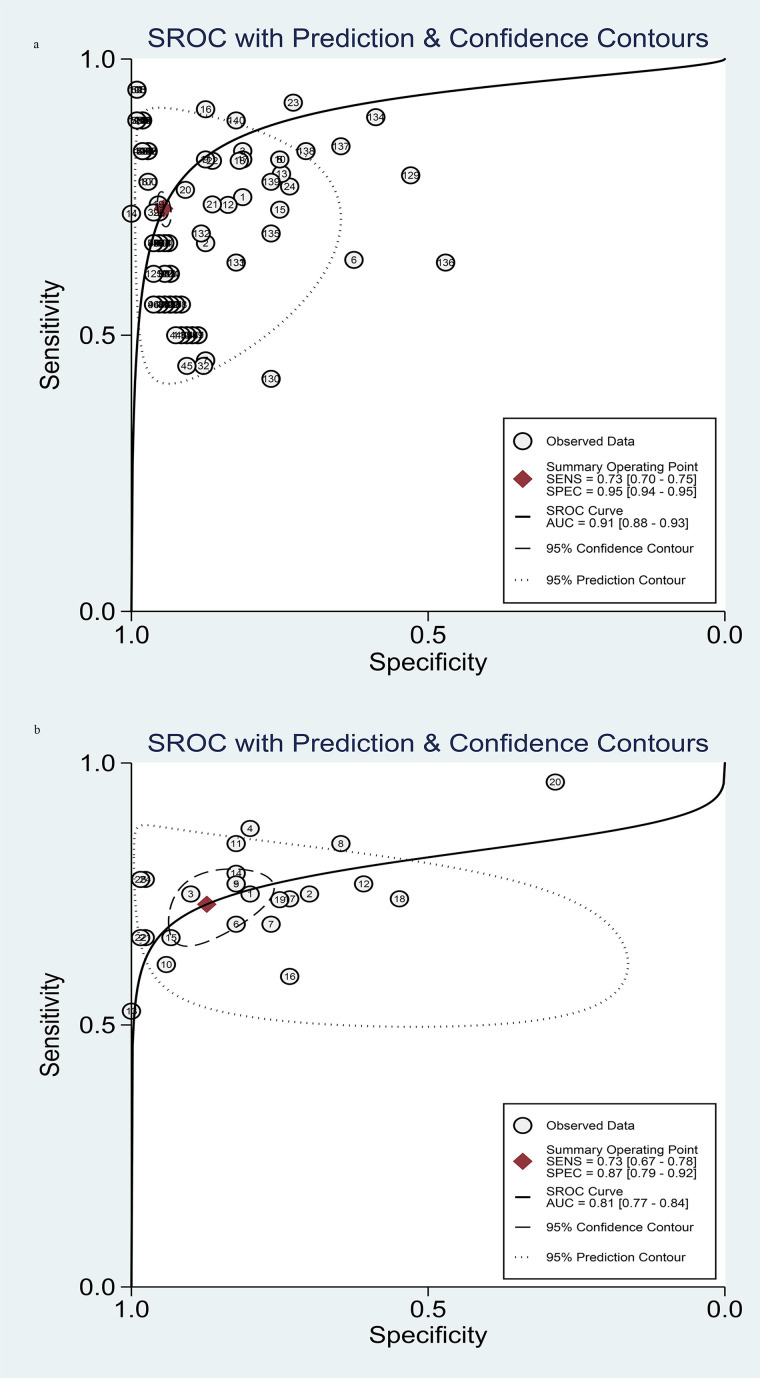
SROC curve with corresponding 95% CIs of the radiomics model in predicting T790M mutation status for non-small cell lung cancer on (a) internal validation and (b) external validation.

The overall radiomics model demonstrated good diagnostic performance for detecting T790M gene mutations in NSCLC. In internal validation, the model demonstrated a pooled AUC of 0.91 (95% CI: 0.88–0.93). Pooled sensitivity was 0.73 (95% CI: 0.70–0.75; *I²* = 50.21%). Pooled specificity was 0.95 (95% CI: 0.94–0.95; *I²* = 81.96%). The summary PLR was 13.40 (95% CI: 11.20–16.10), the NLR was 0.29 (95% CI: 0.26–0.32), and the DOR was 47 (95% CI: 37–60). Performance on external validation cohorts yielded a pooled AUC of 0.81 (95% CI: 0.77–0.84). The summary estimates for sensitivity and specificity were 0.73 (95% CI: 0.67–0.78; *I²* = 55.40%) and 0.87 (95% CI: 0.79–0.92; *I²* = 91.36%), respectively. The PLR was 5.8 (95% CI: 3.6–9.3), the NLR was 0.31 (95% CI: 0.26–0.37), and the DOR was 19 (95% CI: 11–31). The forest plot for pooled sensitivity and specificity is shown in Fig 3. The SROC curves for all studies are depicted in Fig 4.

### Subgroup analysis

The *I*^*2*^ statistic revealed high heterogeneity both in pooled sensitivity (*I²*_*in*_= 50.21%, *I ²*_*ex*_ = 55.40%) and specificity (*I²*_*in*_= 82.96%, *I²*_*ex*_ = 91.36%), indicating greater variability in specificity outcomes and greater consistency in sensitivity and specificity outcomes. We conducted subgroup analyses to identify sources of heterogeneity, with groups adjusted as shown in Tables 3 and 4.

**Table 3:** Results of meta-regression and subgroup analyses of radiological and radiomics model in internal datasets.

Characteristic	Category	Number of datasets	Sensitivity (95% CI)	*P*1	Specificity (95% CI)	*P*2	*P*3
Imaging location	Brain	114	0.71 (0.69 - 0.73)	0.03	0.95 (0.95 - 0.96)	<0.001	<0.001
	Lung	22	0.76 (0.73 - 0.79)		0.81 (0.78 - 0.84)		
	Spine	4	0.73 (0.59 - 0.83)		0.86 (0.75 - 0.92)		
Imaging equipment	MRI	118	0.72 (0.69 - 0.75)	<0.001	0.96 (0.95 - 0.96)	<0.001	<0.001
	CT	22	0.75 (0.69 - 0.80)		0.80 (0.75 - 0.85)		
Segmentation Software	ITK-SNAP	33	0.76 (0.73 - 0.79)	<0.001	0.80 (0.76 - 0.85)	<0.001	0.26
	3D Slicer	3	0.75 (0.70 - 0.80)		0.89 (0.81 - 0.96)		
Reference standard	Blood samples	11	0.74 (0.65-0.81)	0.09	0.81(0.75-0.86)	<0.001	<0.001
	Pathological biopsy	19	0.76 (0.72-0.80)		0.79 (0.75-0.82)		
	Unspecified origin	110	0.71 (0.70-0.73)		0.95 (0.95-0.96)		
RQS	>20	18	0.76 (0.70 - 0.82)	<0.001	0.85 (0.79 - 0.90)	<0.001	<0.001
	≤20	122	0.72 (0.69 - 0.75)		0.95 (0.95 - 0.96)		
Combined clinical parameters	Yes	19	0.79 (0.73 - 0.84)	<0.001	0.81 (0.75 - 0.87)	<0.001	<0.001
	NO	121	0.72 (0.69 - 0.75)		0.95 (0.95 - 0.96)		
Standardization	Yes	19	0.76 (0.69 - 0.82)	<0.001	0.80 (0.73 - 0.86)	<0.001	<0.001
	NO	121	0.72 (0.70 - 0.75)		0.95 (0.95 - 0.96)		

Note: RQS, Radiomics Quality Score.

**Table 4:** Results of meta-regression and subgroup analyses of radiological and radiomics model in external datasets.

Characteristic	Category	Number of studies	Sensitivity (95% CI)	*P*1	Specificity (95% CI)	*P*2	*P*3
Data source	multi-center	5	0.74(0.59 - 0.88)	0.12	0.98 (0.97 - 1.00)	0.14	<0.001
	single-center	20	0.74 (0.69 - 0.80)		0.77 (0.70 - 0.84)		
Imaging location	Brain	13	0.76 (0.71 - 0.80)	0.76	0.70 (0.66 - 0.74)	<0.001	<0.001
	Lung	8	0.73 (0.64 - 0.80)		0.96 (0.95 - 0.98)		
	Spine	4	0.78 (0.61 - 0.89)		0.80 (0.65 - 0.90)		
Imaging equipment	MRI	17	0.75 (0.69 - 0.80)	0.08	0.77 (0.68 - 0.86)	<0.001	<0.001
	CT	8	0.70 (0.59 - 0.80)		0.96 (0.94 - 0.99)		
Segmentation Software	ITK-SNAP	11	0.76 (0.67 - 0.84)	0.04	0.83 (0.70 - 0.95)	0.04	0.55
	3D slicer	14	0.71 (0.65 - 0.78)		0.90 (0.83 - 0.97)		
Reference standard	Blood samples	11	0.76 (0.67-0.84)	0.04	0.83 (0.70-0.95)	0.04	0.55
	Unspecified origin	14	0.71 (0.65-0.78)		0.90 (0.83-0.97)		
RQS	>20	16	0.73 (0.66 - 0.81)	<0.001	0.92 (0.87 - 0.97)	0.80	0.06
	≤20	9	0.75 (0.68 - 0.82)		0.76 (0.61 - 0.91)		
Combined clinical parameters	Yes	3	0.78 (0.60 - 0.95)	0.55	0.97 (0.91 - 1.00)	0.18	0.09
	NO	22	0.73 (0.68 - 0.78)		0.85 (0.78 - 0.92)		
Standardization	Yes	20	0.73 (0.68 - 0.79)	0.11	0.85 (0.77 - 0.93)	0.05	0.34
	NO	5	0.75 (0.60 - 0.89)		0.94 (0.86 - 1.00)		

Note: RQS, Radiomics Quality Score.

In terms of data sources in internal validation, all data were derived from a single-center study. In external validation, single-center studies (n = 20) demonstrated similar sensitivity (0.74 [95% CI, 0.69–0.80] vs 0.74 [95% CI, 0.59–0.88], *P* = 0.12) and lower specificity (0.77 [95% CI, 0.70–0.84] vs 0.98 [95% CI, 0.97–1.00], *P* = 0.14) compared to multicenter studies (n = 5).

By imaging sites in internal validation, imaging examinations for NSCLC in the lungs and mediastinum (n = 22) demonstrated the highest sensitivity (0.76 [95% CI, 0.73–0.79], *P* = 0.03) compared to brain metastases and spinal metastases. However, imaging for brain metastases showed superior specificity (0.95 [95% CI, 0.95–0.96], *P* < 0.001). In external validation, imaging examinations for lung and mediastinal metastases (n = 8) showed superior specificity (0.96 [95% CI, 0.95–0.98], *P* < 0.001).

Regarding imaging modalities, predictive models utilizing MRI imaging (n = 118) demonstrated lower sensitivity (0.72 [95% CI, 0.69–0.75] vs 0.75 [95% CI, 0.69–0.80], *P* < 0.001) and higher specificity (0.96 [95% CI, 0.95–0.96] vs 0.80 [95% CI, 0.75–0.85], *P* < 0.001) compared to those employing CT imaging (n = 22) in internal validation. In external validation, the use of CT imaging (n=8) offered higher sensitivity for fixation (0.96 [95% CI, 0.94–0.99], *P* < 0.001).

In terms of image segmentation software, ITK-SNAP (n = 33) demonstrated higher sensitivity (internal validation, 0.76 [95% CI, 0.73–0.79], *P* < 0.001; external validation, 0.76 [95% CI, 0.67–0.84], *P* = 0.04) and lower specificity (internal: 0.80 [95% CI, 0.76–0.85], *P* < 0.001; external: 0.83 [95% CI, 0.70–0.95], *P* = 0.04).

Regarding the reference standard, in external validation sets, models referenced against blood sample (n=11) achieved higher sensitivity (0.76 [95% CI, 0.67–0.84], *P* = 0.04). Specificity was consistently highest for models employing standards of unspecified origin (internal: 0.95 [95% CI, 0.95–0.96], *P* < 0.001; external: 0.90 [95% CI, 0.83–0.97], *P* = 0.04).

By RQS scores, RQS > 20 (n = 18) demonstrated higher sensitivity (0.76 [95% CI, 0.70–0.82] vs 0.72 [95% CI, 0.69–0.75], *P* < 0.001) and lower specificity (0.85 [95% CI, 0.79–0.90] vs 0.95 [95% CI, 0.95–0.96], *P* < 0.001) compared to RQS ≤ 20 (n = 122) in internal validation. While in external validation, RQS ≤ 20 (n = 9) demonstrated higher sensitivity (0.75 [95% CI, 0.68–0.82], *P* < 0.001).

Studies combining radiomics and clinical factors in internal validation (n = 19) achieved higher sensitivity (0.79 [95% CI, 0.73–0.84], *P* < 0.001) and lower specificity (0.81 [95% CI, 0.75–0.87], *P* < 0.001) compared with studies using radiomics models alone (n = 121).

In internal validation, standardized data processing achieved higher sensitivity (0.76 [95% CI, 0.69–0.82] vs 0.72 [95% CI, 0.70–0.75], *P* < 0.001), but reduced specificity (0.80 [95% CI, 0.73–0.86] vs 0.95 [95% CI, 0.95–0.96], *P* < 0.001).

### Sensitivity analyses

**Fig 5:**
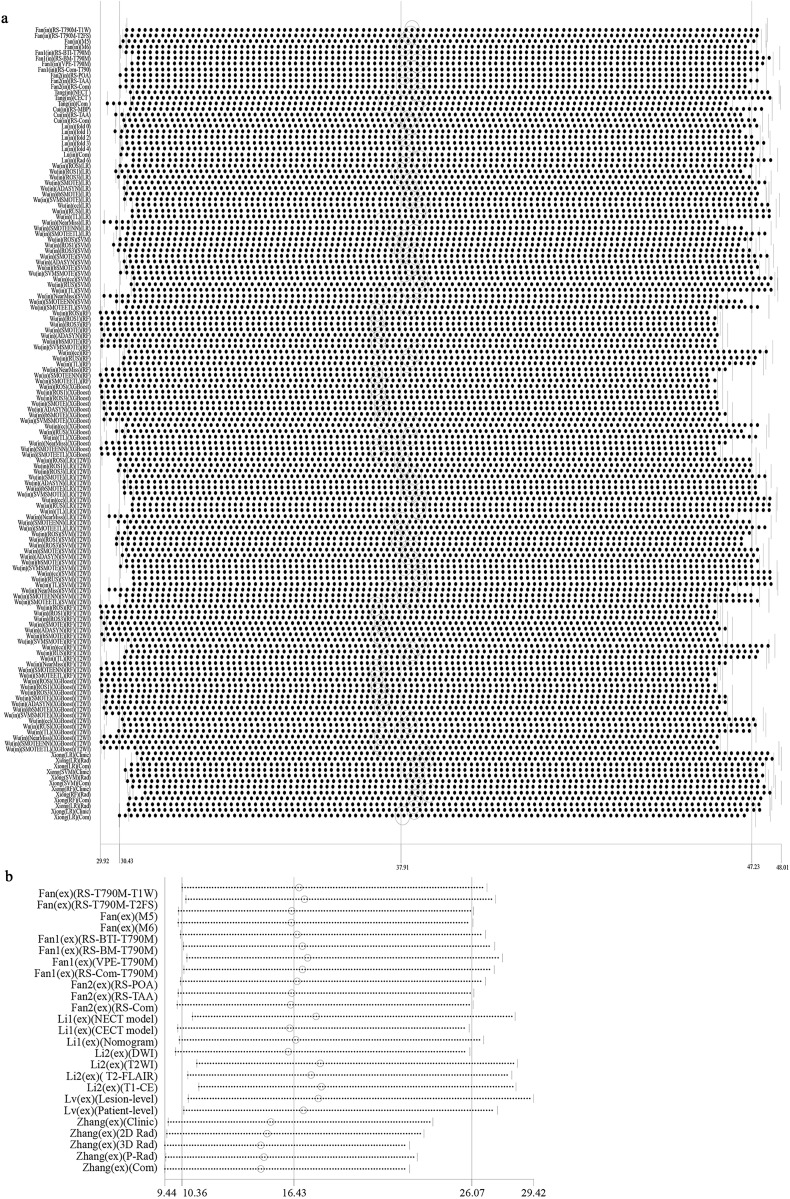
Leave-one-out sensitivity analysis for the diagnostic odds ratio (DOR) on (a) internal validation and (b) external validation.

As shown in Fig 5, sensitivity analysis indicated no significant changes after each systematic exclusion of a study both in internal and external validation.

### Publication bias

**Fig 6:**
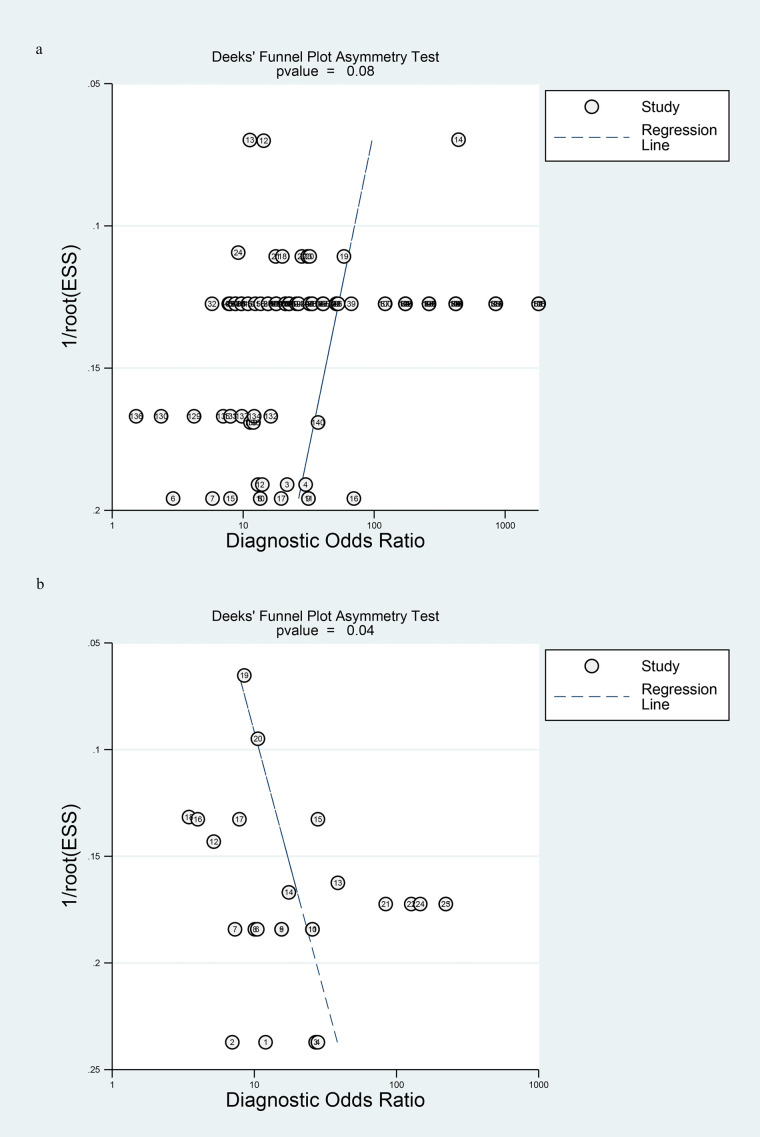
Funnel plot based on the radiomics model in predicting T790M mutation status in non-small cell lung cancer on (a) internal validation and (b) external validation.

The Deeks funnel plot asymmetry test (Fig 6) indicated no significant publication bias for internal validation studies (*P* = 0.08), whereas significant bias was present among external validation studies (*P* = 0.04).

### Predictive values

**Fig 7:**
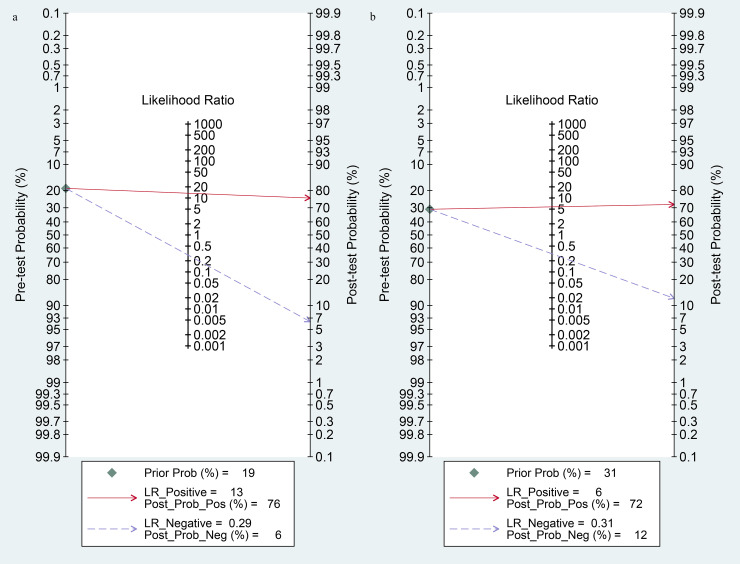
The Fagan nomogram demonstrated the performance of the radiomics model in predicting T790M mutation status for non-small cell lung cancer on (a) internal validation and (b) external validation.

Based on Fagan’s nomogram analysis, in internal validation, applying a radiomics model with a pre-test probability of 19% (When performing diagnostic meta-analysis using the STATA midas command, the software automatically calculates the weighted pooled prevalence based on the raw data from the 2x2 contingency tables (true positives, false positives, etc.) of all included studies) and a PLR of 13.40 increases the post-test probability of T790M expression in NSCLC patients to approximately 76%. Conversely, using an NLR of 0.29 within the same model context reduces the post-test probability to 6%. In external validation, a model with a pre-test probability of 31% and a PLR of 6 elevated the predicted probability to 72%. Conversely, an NLR of 0.31 reduces the posterior positive probability to 12% (Fig 7).

## Discussion

### Radiomics models predict T790M resistance in NSCLC

This study conducted a systematic review and meta-analysis of 13 studies involving 2,654 patients to develop a radiomics-based predictive model for T790M resistance mutations in NSCLC. To our knowledge, this represents the first comprehensive evaluation of radiomics technology for predicting T790M mutations in NSCLC. Pooled results indicate the models demonstrated good performance in predicting T790M mutations (AUCin = 0.91 [0.88–0.93], AUCex = 0.81 [0.77–0.84]), indicating these models can distinguish most T790M mutation cases. The model also demonstrated high specificity (internal validation, 0.95 [95% CI: 0.94–0.95], external validation, 0.87 [95% CI: 0.79–0.92]) and slightly lower sensitivity (internal validation, 0.73 [95% CI: 0.70–0.75], external validation, 0.73 [95% CI: 0.67–0.78]), further highlighting its efficacy. Predicting T790M mutations in NSCLC using radiomics models provides valuable reference for clinical decision-making.

We observed heterogeneity in pooled sensitivity (*I²*_*in*_= 50.21%, *I²*_*ex*_ = 55.40%) and specificity (*I²*_*in*_= 82.962%, *I²*_*ex*_ = 91.36%). This variability can be attributed to multiple methodological factors, including but not limited to: [1] differences in imaging data types and sources; [2] heterogeneity in study populations and mutation status; [3] variations in model construction approaches and feature selection; and [4] discrepancies in study design and clinical context. Fundamentally, the observed heterogeneity reflects a lack of standardization in the technical workflow across studies.

### MRI vs. CT specificity: Varies by body site and validation

MRI and CT, as two distinct imaging modalities, provide insights into different states of NSCLC. However, a critical and confounding factor in our analysis is that MRI and CT studies examined fundamentally different anatomical targets: MRI primarily for brain metastases and CT for thoracic (primary or metastatic) lesions. Therefore, the observed performance differences cannot be attributed to imaging modality alone but are inseparable from the distinct biological and radiological contexts of the anatomical sites. Nevertheless, our exploratory subgroup analysis within internal validation datasets suggests that models focusing on brain metastases (predominantly using MRI) demonstrated exceptionally high specificity (pooled estimate: 0.96) compared to models focusing on thoracic disease (predominantly using CT; pooled estimate: 0.80), albeit with slightly lower sensitivity and high heterogeneity (*I²* > 70%). Brain metastases reside within the unique microenvironment of the central nervous system, where their imaging phenotypes are more heavily modulated by factors such as the blood-brain barrier and local immune environment. This results in more distinctive and consistent imaging alterations produced by T790M-resistant clones, enabling highly specific detection by radiomics models [40,41]. Consequently, MRI models evaluating brain metastases exhibit higher specificity. As the most common primary and metastatic sites for NSCLC, lung and mediastinal lesions are more readily assessed in their entirety on CT. Comprehensive features extracted from these lesions—reflecting tumor texture, shape, and enhancement—can sensitively indicate the presence of T790M-resistant populations. However, the broader range of imaging feature alterations in lung/mediastinal lesions may also incorporate more non-specific changes, resulting in lower specificity compared to MRI models. This performance discrepancy likely reflects heterogeneity in biological behavior and imaging phenotype across anatomical sites [42,43]. Clinically, imaging from specific sites can be prioritized based on practical needs: pursuing high specificity to avoid false positives or prioritizing high sensitivity to reduce missed diagnoses.

Notably, subgroup analyses revealed divergent effects of the same covariates—including imaging modality, anatomical site, and segmentation software—on model sensitivity and specificity between internal and external validation sets. For example, while MRI showed markedly higher specificity than CT in internal validation, CT-based models occasionally achieved higher specificity in external validation, particularly for pulmonary lesions. This discrepancy suggests that performance estimates from internal validation may be inflated owing to cohort homogeneity and model tuning, whereas external validation more accurately reflects generalizability across heterogeneous populations and real-world operational variations. These observations provide a critical framework for interpreting the following technical discussions.

### Models incorporating clinical factors require validation

Integrated models combining radiomics with clinical parameters showed higher pooled sensitivity (0.79 vs. 0.72) but lower specificity (0.81 vs. 0.95) than radiomics-only models. A study by *Fan* et al. in 2022 demonstrated improved predictive capability for detecting EGFR and T790M mutations by combining RS-Coms with smoking status in a developed line chart model [37]. The nomogram yielded higher AUC results, indicating that smoking status complements radiomics features derived from imaging. *Lu* et al. improved prediction of EGFR/T790M mutations by integrating a radiomics score with smoking status in a nomogram [33]. However, *Tang* et al. found that incorporating the Rad-score (which integrates initial EGFR mutation status, EGFR-TKI treatment duration, and CT morphological features-did not further enhance predictive capability [32]. Current research appears to favor including clinical characteristics in radiomics models. Unfortunately, some research indicates that factors such as age, gender, smoking history, and alcohol consumption showed no statistically significant correlation with the timing of T790M emergence [19,20,29,34]. Overall, although some studies suggest that combining clinical features may enhance predictive performance, our subgroup analyses—particularly the internal validation results—indicate that models integrating clinical parameters did not demonstrate numerically significant advantages in the current study. This seemingly contradictory finding may be attributed to differences in the selection of clinical factors across studies and the imaging modalities employed. For instance, we observed that 80% of studies successfully establishing combined clinical feature models utilized CT imaging. In practice, MRI should offer superior predictive value for EGFR mutation status due to its ability to provide richer textural features. Therefore, attempts to incorporate clinical parameters in CT-based studies to compensate for insufficient imaging information may explain the coexistence of the overall conclusion of “no clear advantage for combined models” with individual positive findings.

## Selection of feature segmentation software

In radiomics research, ITK-SNAP and 3D Slicer are the two most commonly used feature segmentation software. Subgroup analysis revealed that ITK-SNAP demonstrated higher sensitivity compared to 3D Slicer (0.76 [95% CI, 0.73–0.79]), while 3D Slicer exhibited greater specificity (0.75 [95% CI, 0.67–0.84] vs.0.81 [95% CI, 0.75–0.88]). This likely stems from differences in image segmentation strategies, feature extraction algorithms, and subsequent processing between the two software packages. ITK-SNAP excels in its semi-automated segmentation capability, which initiates segmentation from seed points and includes surrounding voxels based on intensity. In cases of ambiguous grayscale transitions, this may result in over-segmentation and subsequent volume overestimation. This approach may offer advantages in capturing the overall tumor morphology and internal heterogeneity. Even when operators strive for precise boundaries, the algorithm’s characteristics may cause it to favor inclusion of peritumoral areas suspected of reaction or infiltration. This aids in capturing more potential imaging signals associated with EGFR mutations (including subtle or diffuse textural alterations), thereby enhancing the ability to detect true positives. However, it may also introduce non-specific features or noise, such as including small amounts of non-tumor tissue. These features may be misclassified as positive by the model, potentially reducing specificity. While 3D Slicer supports multiple segmentation methods, manual layer-by-layer delineation remains a common approach in many studies. When manually delineating, operators may adhere more strictly to clearly defined tumor boundaries in the images, favoring the acquisition of purer intratumoral features. This approach reduces the influence of peritumoral tissue or uncertain regions, potentially yielding more specific features that better represent characteristics unique to EGFR mutations. Consequently, it may help reduce false positives and improve specificity. Conversely, it may overlook some valid biological signals located at the tumor margin or peritumorally, leading to the omission of some true positive cases and potentially reducing sensitivity [44].

### Impact of reference standard on performance estimates

Based on our subgroup analysis, the reference standard significantly influenced performance estimates, with a key methodological constraint: no studies in the external validation sets utilized a definitive tissue biopsy reference standard. Within this limited analytical context, models referenced against plasma ctDNA demonstrated notable sensitivity (0.76, 95% CI: 0.67–0.84). This finding may be exploratory, suggesting that in heterogeneous external cohorts, the systemic nature of liquid biopsy might align differently with radiomic phenotypes compared to other non-tissue standards. It does not imply superiority over a tissue-based benchmark, which was absent for direct comparison. Critically, models employing an unspecified reference standard consistently yielded the highest pooled specificity (internal: 0.95; external: 0.90), most plausibly reflecting a systematic overestimation bias. As tissue biopsy remains the clinical gold standard for spatial specificity and lower false-negative rates, the absence of tissue-validated models in external validation represents a major evidence gap. Therefore, the performance of plasma-based standards should be interpreted as preliminary. Future studies must prioritize external validation with histologically confirmed cohorts to establish unbiased, clinically relevant performance benchmarks.

### Impact of radiomics workflow on performance

The exploratory subgroup analysis based on the RQS revealed an association between methodological quality and the generalizability of model performance. It is crucial to emphasize that the RQS evaluates methodological rigor, not absolute performance [45]. We observed that studies with higher RQS tended to report more conservative sensitivity in internal validation, while their models demonstrated more robust preservation of specificity in external validation. This pattern may be explained by a mitigation of overfitting through stringent methodology. Higher-quality studies typically employ more rigorous feature selection and validation strategies, resulting in internal performance estimates—particularly for sensitivity—that are less optimistically biased and closer to a model’s true generalizable capability [46]. Consequently, when applied to independent external data, these models exhibit less performance degradation, with specificity proving particularly resilient. This indicates that methodological rigor does not merely lower reported metrics but yields models with more reliable and reproducible performance profiles, especially regarding the sensitivity-specificity trade-off across different populations. This underscores the necessity of adhering to high methodological standards to develop radiomic models that can be trusted in broader clinical practice.

Model validation methods include internal validation and external validation. Currently, for developed radiomics models, most evaluate predictive performance through internal validation. According to the literature, external validation is recommended for datasets exceeding 50 samples, while re-validation methods are recommended for smaller datasets [47]. The AUC values from external validation appeared lower than those from internal validation or the training cohort, consistent with characteristics observed in prior studies. It is worth noting that most of the studies we included were concentrated in China. This may be because cancer treatment in China often occurs at large regional medical centers specializing in oncology, which facilitates data retention and thus leads to a higher concentration of patients. This also suggests the need for more studies from other regions to reduce bias.

Our findings support the use of radiomics as a potential screening tool for determining the T790M mutation status in NSCLC and align with those of *Fuster-Matanzo* et al., who conducted a meta-analysis of 124 studies on the diagnostic performance of radiomics technology for predicting oncogene mutation status of NSCLC, reporting an AUC of 0.821 and a sensitivity of 0.806 (95% CI 0.776–0.833) in EGFR, respectively [48]. Imaging modalities should be selected based on the patient’s disease stage. CT is widely used in advanced NSCLC, while MRI is extensively employed for brain and bone metastases. Finally, future studies should prioritize additional validation and consider critical aspects, primarily ensuring minimum sample sizes to guarantee the reliability of results obtained using artificial intelligence (AI)-based models. In our systematic review and meta-analysis, over half (11/13) of the validation cohorts had sample sizes >30, with only two studies having a smaller sample size, which may limit the relevance of the conclusions. Although multicenter designs enhance the reliability of conclusions, only one study employed this approach. A small number of studies used validation data from different centers for internal and external validation, respectively. These findings complement our efforts to minimize bias in meta-analyses and underscore the importance of comprehensive and transparent reporting in future research.

### Limitation

This study has several limitations that may affect the interpretation and generalizability of the results. First, the number and quality of studies available for meta-analysis were limited. Although AI algorithms have received extensive attention in predicting T790M gene mutations in NSCLC, related studies may still be relatively scarce. This may be because the application of AI algorithms is still in the developmental stage, and relevant research is underway. Additionally, differences among studies-including variations in study design, sample size, data collection, and evaluation methods-contributed to result heterogeneity. The generalizability of our findings is constrained by the exclusive inclusion of studies conducted in China. This geographic homogeneity limits the assessment of how variations in *EGFR* mutation epidemiology, imaging protocols, and clinical workflows across global populations and healthcare systems may affect model performance. The observed heterogeneity underscores this concern and highlights the critical need for external validation in multinational cohorts to confirm clinical utility. Retrospective studies carry recall bias, necessitating prospective studies that encompass broader geographic regions to validate our conclusions. Second, different data sources, acquisition methods, image quality, and feature extraction techniques may have been used across the studies identified through literature searches. Additionally, this study did not directly compare radiomics models with existing diagnostic strategies such as liquid biopsy, nor did it evaluate their net benefit in clinical decision-making through decision curve analysis. This represents a significant limitation of the research. Our exploratory subgroup analyses (not pre-specified) suggest differences across imaging modalities, anatomical sites, and software tools, but these may be confounded by non-randomized allocations, small sample sizes, and heterogeneity. Hence, they should be viewed as hypothesis-generating rather than definitive. In this light, radiomics is best positioned as a non-invasive, reproducible supplementary tool to guide decisions when biopsy is unsuitable; however, its true clinical utility—including effects on patient outcomes—requires prospective validation with head-to-head comparisons against standard strategies and formal health economic analyses.

## Conclusion

In conclusion, radiomics demonstrates technical promise as a non-invasive screening tool for predicting T790M mutation status in NSCLC based on retrospective evidence. However, it is essential to recognize that these imaging-based models are intended to complement, rather than replace, confirmatory tissue biopsy, which remains the diagnostic gold standard. The universal absence of prospective and clinically validated studies precludes conclusions about immediate clinical utility. Further comprehensive and prospective multicenter investigations are needed to validate these findings and facilitate the clinical integration of AI-driven imaging in this field.

## Supporting information

S1 Table PRISMA checklist of this meta-analysis. (DOCX)

S2 Table Search strategy. (DOCX)

S3 Table The table of radiomics quality scores 2.0 for each study. (DOCX)

S4 Table The fundamental characteristics incorporated into the validation model. (DOCX)

S1 File The review protocol. (DOCX)
