# Supplementary material for: Predicting T790M mutation status in non-small cell lung cancer based on radiomics: A systematic review and meta-analysis
Source: PLoS One. 2026 Jul 8;21(7):e0353257. doi: 10.1371/journal.pone.0353257 (PMC13345267; doi:10.1371/journal.pone.0353257)
Supplement: S2 Table — (DOCX) [file pone.0353257.s002.docx]

**Supplementary Table 2.Search Strategy**

| Search Terms |
| --- |
| Based on PubMed, Embase, Web of Science databases, China National Knowledge Infrastructure, and Wanfang. |
| ((Pulmonary Cancer*[Title/Abstract])OR (Pulmonary Neoplasm*[Title/Abstract])OR (Lung Neoplasm*[Title/Abstract])OR (Lung Cancer*[Title/Abstract])OR (Neoplasm of the lung[Title/Abstract])OR (Neoplasm of lung[Title/Abstract])OR (Cancer of Lung[Title/Abstract])OR (carcinoma of the lung[Title/Abstract])OR (lung carcinoma[Title/Abstract])OR (Cancer of the Lung[Title/Abstract]))AND ((CT[Title/Abstract])OR (MRI[Title/Abstract])OR (PET-CT[Title/Abstract])OR (radiomic[Title/Abstract])OR (imaging[Title/Abstract]))AND ((EGFR[Title/Abstract])) |
