# Supplementary material for: Predicting T790M mutation status in non-small cell lung cancer based on radiomics: A systematic review and meta-analysis
Source: PLoS One. 2026 Jul 8;21(7):e0353257. doi: 10.1371/journal.pone.0353257 (PMC13345267; doi:10.1371/journal.pone.0353257)
Supplement: S4 Table — (DOCX) [file pone.0353257.s004.docx]

**Supplementary Table 4.** **The fundamental characteristics incorporated into the validation model.**

| Author | Year | Country | Sensitivity | Specificity | AUC | Data source | External validation | Imaging location | Imaging equipment | Segmentation Software | RQS | Combined clinical parameters | Standardization | Reference Standard |
| --- | --- | --- | --- | --- | --- | --- | --- | --- | --- | --- | --- | --- | --- | --- |
| Fan(in)(RS-T790M-T1W) | 2022 | China | 0.75 | 0.81 | 0.80 | single center | internal validation | Spine | MRI | ITK-SNAP | 21 | No | No | blood samples |
| Fan(in)(RS-T790M-T2FS) | 2022 | China | 0.67 | 0.88 | 0.76 | single center | internal validation | Spine | MRI | ITK-SNAP | 21 | No | No | blood samples |
| Fan(in)(M5) | 2022 | China | 0.83 | 0.81 | 0.81 | single center | internal validation | Spine | MRI | ITK-SNAP | 21 | No | No | blood samples |
| Fan(in)(M6) | 2022 | China | 0.67 | 0.94 | 0.82 | single center | internal validation | Spine | MRI | ITK-SNAP | 21 | Yes | No | blood samples |
| Fan(ex)(RS-T790M-T1W) | 2022 | China | 0.75 | 0.80 | 0.73 | single center | external validation | Spine | MRI | ITK-SNAP | 21 | No | No | blood samples |
| Fan(ex)(RS-T790M-T2FS) | 2022 | China | 0.75 | 0.70 | 0.71 | single center | external validation | Spine | MRI | ITK-SNAP | 21 | No | No | blood samples |
| Fan(ex)(M5) | 2022 | China | 0.75 | 0.90 | 0.78 | single center | external validation | Spine | MRI | ITK-SNAP | 21 | No | No | blood samples |
| Fan(ex)(M6) | 2022 | China | 0.88 | 0.80 | 0.80 | single center | external validation | Spine | MRI | ITK-SNAP | 21 | Yes | No | blood samples |
| Fan1(in)(RS-BTI-T790M) | 2023 | China | 0.77 | 0.73 | 0.81 | single center | internal validation | Brain metastases | MRI | ITK-SNAP | 21 | No | Yes | blood samples |
| Fan1(in)(RS-BM-T790M) | 2023 | China | 0.68 | 0.64 | 0.69 | single center | internal validation | Brain metastases | MRI | ITK-SNAP | 21 | No | Yes | blood samples |
| Fan1(ex)(RS-BTI-T790M) | 2023 | China | 0.77 | 0.85 | 0.65 | single center | external validation | Brain metastases | MRI | ITK-SNAP | 21 | No | Yes | blood samples |
| Fan1(ex)(RS-BM-T790M) | 2023 | China | 0.67 | 0.85 | 0.59 | single center | external validation | Brain metastases | MRI | ITK-SNAP | 21 | No | Yes | blood samples |
| Fan1(in)(VPE-T790M) | 2023 | China | 0.46 | 0.88 | 0.57 | single center | internal validation | Brain metastases | MRI | ITK-SNAP | 21 | No | Yes | blood samples |
| Fan1(in)(RS-Com-T790) | 2023 | China | 0.82 | 0.75 | 0.79 | single center | internal validation | Brain metastases | MRI | ITK-SNAP | 21 | No | Yes | blood samples |
| Fan1(ex)(VPE-T790M) | 2023 | China | 0.69 | 0.77 | 0.61 | single center | external validation | Brain metastases | MRI | ITK-SNAP | 21 | No | Yes | blood samples |
| Fan1(ex)(RS-Com-T790) | 2023 | China | 0.85 | 0.65 | 0.77 | single center | external validation | Brain metastases | MRI | ITK-SNAP | 21 | No | Yes | blood samples |
| Fan2(in)(RS-POA) | 2023 | China | 0.82 | 0.88 | 0.81 | single center | internal validation | Brain, POA, and TAA | MRI | ITK-SNAP | 19 | No | Yes | blood samples |
| Fan2(in)(RS-TAA) | 2023 | China | 0.82 | 0.75 | 0.81 | single center | internal validation | Brain, POA, and TAA | MRI | ITK-SNAP | 19 | No | Yes | blood samples |
| Fan2(in)(RS-Com) | 2023 | China | 0.82 | 0.88 | 0.86 | single center | internal validation | Brain, POA, and TAA | MRI | ITK-SNAP | 19 | No | Yes | blood samples |
| Fan2(ex)(RS-POA) | 2023 | China | 0.77 | 0.82 | 0.81 | single center | external validation | Brain, POA, and TAA | MRI | ITK-SNAP | 19 | No | Yes | blood samples |
| Fan2(ex)(RS-TAA) | 2023 | China | 0.62 | 0.94 | 0.78 | single center | external validation | Brain, POA, and TAA | MRI | ITK-SNAP | 19 | No | Yes | blood samples |
| Fan2(ex)(RS-Com) | 2023 | China | 0.85 | 0.82 | 0.86 | single center | external validation | Brain, POA, and TAA | MRI | ITK-SNAP | 19 | No | Yes | blood samples |
| Li1(ex)(NECT model) | 2023 | China | 0.78 | 0.60 | 0.62 | single center | external validation | Lung and mediastinum | NECT | 3D slicer | 21 | No | Yes | genetic test reports |
| Li1(ex)(CECT model) | 2023 | China | 0.53 | 1.00 | 0.75 | single center | external validation | Lung and mediastinum | CECT | 3D slicer | 21 | No | Yes | genetic test reports |
| Li1(ex)(Nomogram) | 2023 | China | 0.80 | 0.80 | 0.85 | single center | external validation | Lung and mediastinum | NECT+CECT | 3D slicer | 21 | No | Yes | genetic test reports |
| Li2(ex)(DWI) | 2023 | China | 0.65 | 0.93 | 0.85 | single center | external validation | Brain metastases | MRI | 3D Slicer | 18 | No | Yes | pathological biopsy or blood samples |
| Li2(ex)(T2WI) | 2023 | China | 0.60 | 0.73 | 0.73 | single center | external validation | Brain metastases | MRI | 3D Slicer | 18 | No | Yes | pathological biopsy or blood samples |
| Li2(ex)( T2-FLAIR) | 2023 | China | 0.73 | 0.73 | 0.81 | single center | external validation | Brain metastases | MRI | 3D Slicer | 18 | No | Yes | pathological biopsy or blood samples |
| Li2(ex)(T1-CE) | 2023 | China | 0.75 | 0.55 | 0.73 | single center | external validation | Brain metastases | MRI | 3D Slicer | 18 | No | Yes | pathological biopsy or blood samples |
| Lv(ex)(Lesion-level) | 2023 | China | 0.74 | 0.75 | 0.83 | single center | external validation | Brain metastases | MRI | 3D Slicer | 19 | No | Yes | pathological biopsy or blood samples |
| Lv(ex)(Patient-level) | 2023 | China | 0.96 | 0.29 | 0.73 | single center | external validation | Brain metastases | MRI | 3D Slicer | 19 | No | Yes | pathological biopsy or blood samples |
| Tang(in)(NECT ) | 2023 | China | 0.74 | 0.84 | 0.84 | single center | internal validation | Lung and mediastinum | NECT | 3D Slicer | 25 | No | Yes | pathological biopsy or blood samples |
| Tang(in)(CECT ) | 2023 | China | 0.79 | 0.75 | 0.81 | single center | internal validation | Lung and mediastinum | CECT | 3D Slicer | 25 | No | Yes | pathological biopsy or blood samples |
| Tang(in)(Com ) | 2023 | China | 0.72 | 1.00 | 0.90 | single center | internal validation | Lung and mediastinum | NECT+CECT | 3D Slicer | 25 | No | Yes | pathological biopsy or blood samples |
| Cui(in)(RS-MBP) | 2024 | China | 0.73 | 0.78 | 0.79 | single center | internal validation | Brain metastases | MRI | ITK-SNAP | 18 | Yes | No | genetic test reports |
| Cui(in)(RS-TAA) | 2024 | China | 0.91 | 0.91 | 0.78 | single center | internal validation | Brain metastases | MRI | ITK-SNAP | 18 | Yes | No | genetic test reports |
| Cui(in)(RS-Com) | 2024 | China | 0.82 | 0.81 | 0.82 | single center | internal validation | Brain metastases | MRI | ITK-SNAP | 18 | Yes | No | genetic test reports |
| Lu(in)(fold 0) | 2024 | China | 0.82 | 0.81 | 0.83 | single center | internal validation | Lung and mediastinum | CT | ITK-SNAP | 22 | Yes | Yes | pathological biopsy |
| Lu(in)(fold 1) | 2024 | China | 0.73 | 0.95 | 0.87 | single center | internal validation | Lung and mediastinum | CT | ITK-SNAP | 22 | Yes | Yes | pathological biopsy |
| Lu(in)(fold 2) | 2024 | China | 0.77 | 0.91 | 0.86 | single center | internal validation | Lung and mediastinum | CT | ITK-SNAP | 22 | Yes | Yes | pathological biopsy |
| Lu(in)(fold 3) | 2024 | China | 0.73 | 0.86 | 0.84 | single center | internal validation | Lung and mediastinum | CT | ITK-SNAP | 22 | Yes | Yes | pathological biopsy |
| Lu(in)(fold 4) | 2024 | China | 0.82 | 0.86 | 0.88 | single center | internal validation | Lung and mediastinum | CT | ITK-SNAP | 22 | Yes | Yes | pathological biopsy |
| Lu(in)(Com) | 2024 | China | 0.91 | 0.73 | 0.87 | single center | internal validation | Lung and mediastinum | CT | ITK-SNAP | 22 | Yes | Yes | pathological biopsy |
| Lu(in)(Rad 6) | 2024 | China | 0.78 | 0.73 | 0.80 | single center | internal validation | Lung and mediastinum | CT | ITK-SNAP | 22 | Yes | Yes | pathological biopsy |
| Wu(in)(ROS)(LR) | 2024 | China and the USA | 0.67 | 0.95 | 0.70 | single center | internal validation | Brain metastases | MRI | NA | 20 | No | No | genetic test reports |
| Wu(in)(ROS1)(LR) | 2024 | China and the USA | 0.71 | 0.96 | 0.70 | single center | internal validation | Brain metastases | MRI | NA | 20 | No | No | genetic test reports |
| Wu(in)(ROS3)(LR) | 2024 | China and the USA | 0.69 | 0.96 | 0.69 | single center | internal validation | Brain metastases | MRI | NA | 20 | No | No | genetic test reports |
| Wu(in)(SMOTE)(LR) | 2024 | China and the USA | 0.53 | 0.92 | 0.56 | single center | internal validation | Brain metastases | MRI | NA | 20 | No | No | genetic test reports |
| Wu(in)(ADASYN)(LR) | 2024 | China and the USA | 0.59 | 0.93 | 0.64 | single center | internal validation | Brain metastases | MRI | NA | 20 | No | No | genetic test reports |
| Wu(in)(bSMOTE)(LR) | 2024 | China and the USA | 0.58 | 0.93 | 0.62 | single center | internal validation | Brain metastases | MRI | NA | 20 | No | No | genetic test reports |
| Wu(in)(SVMSMOTE)(LR) | 2024 | China and the USA | 0.57 | 0.94 | 0.62 | single center | internal validation | Brain metastases | MRI | NA | 20 | No | No | genetic test reports |
| Wu(in)(cc)(LR) | 2024 | China and the USA | 0.43 | 0.88 | 0.40 | single center | internal validation | Brain metastases | MRI | NA | 20 | No | No | genetic test reports |
| Wu(in)(RUS)(LR) | 2024 | China and the USA | 0.49 | 0.90 | 0.48 | single center | internal validation | Brain metastases | MRI | NA | 20 | No | No | genetic test reports |
| Wu(in)(TL)(LR) | 2024 | China and the USA | 0.51 | 0.91 | 0.52 | single center | internal validation | Brain metastases | MRI | NA | 20 | No | No | genetic test reports |
| Wu(in)(NearMiss)(LR) | 2024 | China and the USA | 0.83 | 0.98 | 0.87 | single center | internal validation | Brain metastases | MRI | NA | 20 | No | No | genetic test reports |
| Wu(in)(SMOTEENN)(LR) | 2024 | China and the USA | 0.67 | 0.96 | 0.77 | single center | internal validation | Brain metastases | MRI | NA | 20 | No | No | genetic test reports |
| Wu(in)(SMOTEETL)(LR) | 2024 | China and the USA | 0.53 | 0.92 | 0.60 | single center | internal validation | Brain metastases | MRI | NA | 20 | No | No | genetic test reports |
| Wu(in)(ROS)(SVM) | 2024 | China and the USA | 0.67 | 0.95 | 0.71 | single center | internal validation | Brain metastases | MRI | NA | 20 | No | No | genetic test reports |
| Wu(in)(ROS1)(SVM) | 2024 | China and the USA | 0.72 | 0.96 | 0.73 | single center | internal validation | Brain metastases | MRI | NA | 20 | No | No | genetic test reports |
| Wu(in)(ROS3)(SVM) | 2024 | China and the USA | 0.66 | 0.95 | 0.69 | single center | internal validation | Brain metastases | MRI | NA | 20 | No | No | genetic test reports |
| Wu(in)(SMOTE)(SVM) | 2024 | China and the USA | 0.52 | 0.92 | 0.58 | single center | internal validation | Brain metastases | MRI | NA | 20 | No | No | genetic test reports |
| Wu(in)(ADASYN)(SVM) | 2024 | China and the USA | 0.60 | 0.94 | 0.66 | single center | internal validation | Brain metastases | MRI | NA | 20 | No | No | genetic test reports |
| Wu(in)(bSMOTE)(SVM) | 2024 | China and the USA | 0.57 | 0.93 | 0.63 | single center | internal validation | Brain metastases | MRI | NA | 20 | No | No | genetic test reports |
| Wu(in)(SVMSMOTE)(SVM) | 2024 | China and the USA | 0.56 | 0.96 | 0.62 | single center | internal validation | Brain metastases | MRI | NA | 20 | No | No | genetic test reports |
| Wu(in)(cc)(SVM) | 2024 | China and the USA | 0.45 | 0.91 | 0.46 | single center | internal validation | Brain metastases | MRI | NA | 20 | No | No | genetic test reports |
| Wu(in)(RUS)(SVM) | 2024 | China and the USA | 0.50 | 0.91 | 0.49 | single center | internal validation | Brain metastases | MRI | NA | 20 | No | No | genetic test reports |
| Wu(in)(TL)(SVM) | 2024 | China and the USA | 0.50 | 0.88 | 0.46 | single center | internal validation | Brain metastases | MRI | NA | 20 | No | No | genetic test reports |
| Wu(in)(NearMiss)(SVM) | 2024 | China and the USA | 0.84 | 0.98 | 0.86 | single center | internal validation | Brain metastases | MRI | NA | 20 | No | No | genetic test reports |
| Wu(in)(SMOTEENN)(SVM) | 2024 | China and the USA | 0.67 | 0.96 | 0.76 | single center | internal validation | Brain metastases | MRI | NA | 20 | No | No | genetic test reports |
| Wu(in)(SMOTEETL)(SVM) | 2024 | China and the USA | 0.55 | 0.93 | 0.63 | single center | internal validation | Brain metastases | MRI | NA | 20 | No | No | genetic test reports |
| Wu(in)(ROS)(RF) | 2024 | China and the USA | 0.95 | 0.99 | 0.99 | single center | internal validation | Brain metastases | MRI | NA | 20 | No | No | genetic test reports |
| Wu(in)(ROS1)(RF) | 2024 | China and the USA | 0.90 | 0.99 | 0.91 | single center | internal validation | Brain metastases | MRI | NA | 20 | No | No | genetic test reports |
| Wu(in)(ROS3)(RF) | 2024 | China and the USA | 0.91 | 0.99 | 0.91 | single center | internal validation | Brain metastases | MRI | NA | 20 | No | No | genetic test reports |
| Wu(in)(SMOTE)(RF) | 2024 | China and the USA | 0.87 | 0.98 | 0.92 | single center | internal validation | Brain metastases | MRI | NA | 20 | No | No | genetic test reports |
| Wu(in)(ADASYN)(RF) | 2024 | China and the USA | 0.86 | 0.98 | 0.94 | single center | internal validation | Brain metastases | MRI | NA | 20 | No | No | genetic test reports |
| Wu(in)(bSMOTE)(RF) | 2024 | China and the USA | 0.88 | 0.98 | 0.92 | single center | internal validation | Brain metastases | MRI | NA | 20 | No | No | genetic test reports |
| Wu(in)(SVMSMOTE)(RF) | 2024 | China and the USA | 0.88 | 0.98 | 0.93 | single center | internal validation | Brain metastases | MRI | NA | 20 | No | No | genetic test reports |
| Wu(in)(cc)(RF) | 2024 | China and the USA | 0.55 | 0.92 | 0.55 | single center | internal validation | Brain metastases | MRI | NA | 20 | No | No | genetic test reports |
| Wu(in)(RUS)(RF) | 2024 | China and the USA | 0.60 | 0.94 | 0.55 | single center | internal validation | Brain metastases | MRI | NA | 20 | No | No | genetic test reports |
| Wu(in)(TL)(RF) | 2024 | China and the USA | 0.56 | 0.95 | 0.72 | single center | internal validation | Brain metastases | MRI | NA | 20 | No | No | genetic test reports |
| Wu(in)(NearMiss)(RF) | 2024 | China and the USA | 0.81 | 0.97 | 0.88 | single center | internal validation | Brain metastases | MRI | NA | 20 | No | No | genetic test reports |
| Wu(in)(SMOTEENN)(RF) | 2024 | China and the USA | 0.93 | 0.99 | 0.98 | single center | internal validation | Brain metastases | MRI | NA | 20 | No | No | genetic test reports |
| Wu(in)(SMOTEETL)(RF) | 2024 | China and the USA | 0.89 | 0.98 | 0.95 | single center | internal validation | Brain metastases | MRI | NA | 20 | No | No | genetic test reports |
| Wu(in)(ROS)(XGBoost) | 2024 | China and the USA | 0.93 | 0.99 | 0.96 | single center | internal validation | Brain metastases | MRI | NA | 20 | No | No | genetic test reports |
| Wu(in)(ROS1)(XGBoost) | 2024 | China and the USA | 0.88 | 0.98 | 0.91 | single center | internal validation | Brain metastases | MRI | NA | 20 | No | No | genetic test reports |
| Wu(in)(ROS3)(XGBoost) | 2024 | China and the USA | 0.90 | 0.99 | 0.92 | single center | internal validation | Brain metastases | MRI | NA | 20 | No | No | genetic test reports |
| Wu(in)(SMOTE)(XGBoost) | 2024 | China and the USA | 0.85 | 0.98 | 0.90 | single center | internal validation | Brain metastases | MRI | NA | 20 | No | No | genetic test reports |
| Wu(in)(ADASYN)(XGBoost) | 2024 | China and the USA | 0.86 | 0.98 | 0.90 | single center | internal validation | Brain metastases | MRI | NA | 20 | No | No | genetic test reports |
| Wu(in)(bSMOTE)(XGBoost) | 2024 | China and the USA | 0.85 | 0.97 | 0.89 | single center | internal validation | Brain metastases | MRI | NA | 20 | No | No | genetic test reports |
| Wu(in)(SVMSMOTE)(XGBoost) | 2024 | China and the USA | 0.86 | 0.98 | 0.89 | single center | internal validation | Brain metastases | MRI | NA | 20 | No | No | genetic test reports |
| Wu(in)(cc)(XGBoost) | 2024 | China and the USA | 0.58 | 0.93 | 0.47 | single center | internal validation | Brain metastases | MRI | NA | 20 | No | No | genetic test reports |
| Wu(in)(RUS)(XGBoost) | 2024 | China and the USA | 0.67 | 0.96 | 0.68 | single center | internal validation | Brain metastases | MRI | NA | 20 | No | No | genetic test reports |
| Wu(in)(TL)(XGBoost) | 2024 | China and the USA | 0.57 | 0.94 | 0.64 | single center | internal validation | Brain metastases | MRI | NA | 20 | No | No | genetic test reports |
| Wu(in)(NearMiss)(XGBoost) | 2024 | China and the USA | 0.83 | 0.97 | 0.83 | single center | internal validation | Brain metastases | MRI | NA | 20 | No | No | genetic test reports |
| Wu(in)(SMOTEENN)(XGBoost) | 2024 | China and the USA | 0.91 | 0.99 | 0.95 | single center | internal validation | Brain metastases | MRI | NA | 20 | No | No | genetic test reports |
| Wu(in)(SMOTEETL)(XGBoost) | 2024 | China and the USA | 0.88 | 0.98 | 0.94 | single center | internal validation | Brain metastases | MRI | NA | 20 | No | No | genetic test reports |
| Wu(in)(ROS)(LR)(T2WI) | 2024 | China and the USA | 0.68 | 0.95 | 0.72 | single center | internal validation | Brain metastases | MRI | NA | 20 | No | No | genetic test reports |
| Wu(in)(ROS1)(LR)(T2WI) | 2024 | China and the USA | 0.66 | 0.95 | 0.61 | single center | internal validation | Brain metastases | MRI | NA | 20 | No | No | genetic test reports |
| Wu(in)(ROS3)(LR)(T2WI) | 2024 | China and the USA | 0.66 | 0.95 | 0.57 | single center | internal validation | Brain metastases | MRI | NA | 20 | No | No | genetic test reports |
| Wu(in)(SMOTE)(LR)(T2WI) | 2024 | China and the USA | 0.56 | 0.93 | 0.59 | single center | internal validation | Brain metastases | MRI | NA | 20 | No | No | genetic test reports |
| Wu(in)(ADASYN)(LR)(T2WI) | 2024 | China and the USA | 0.61 | 0.94 | 0.67 | single center | internal validation | Brain metastases | MRI | NA | 20 | No | No | genetic test reports |
| Wu(in)(bSMOTE)(LR)(T2WI) | 2024 | China and the USA | 0.60 | 0.93 | 0.63 | single center | internal validation | Brain metastases | MRI | NA | 20 | No | No | genetic test reports |
| Wu(in)(SVMSMOTE)(LR)(T2WI) | 2024 | China and the USA | 0.57 | 0.95 | 0.62 | single center | internal validation | Brain metastases | MRI | NA | 20 | No | No | genetic test reports |
| Wu(in)(cc)(LR)(T2WI) | 2024 | China and the USA | 0.49 | 0.90 | 0.46 | single center | internal validation | Brain metastases | MRI | NA | 20 | No | No | genetic test reports |
| Wu(in)(RUS)(LR)(T2WI) | 2024 | China and the USA | 0.48 | 0.91 | 0.50 | single center | internal validation | Brain metastases | MRI | NA | 20 | No | No | genetic test reports |
| Wu(in)(TL)(LR)(T2WI) | 2024 | China and the USA | 0.52 | 0.91 | 0.59 | single center | internal validation | Brain metastases | MRI | NA | 20 | No | No | genetic test reports |
| Wu(in)(NearMiss)(LR)(T2WI) | 2024 | China and the USA | 0.79 | 0.97 | 0.87 | single center | internal validation | Brain metastases | MRI | NA | 20 | No | No | genetic test reports |
| Wu(in)(SMOTEENN)(LR)(T2WI) | 2024 | China and the USA | 0.65 | 0.95 | 0.73 | single center | internal validation | Brain metastases | MRI | NA | 20 | No | No | genetic test reports |
| Wu(in)(SMOTEETL)(LR)(T2WI) | 2024 | China and the USA | 0.55 | 0.93 | 0.63 | single center | internal validation | Brain metastases | MRI | NA | 20 | No | No | genetic test reports |
| Wu(in)(ROS)(SVM)(T2WI) | 2024 | China and the USA | 0.68 | 0.95 | 0.73 | single center | internal validation | Brain metastases | MRI | NA | 20 | No | No | genetic test reports |
| Wu(in)(ROS1)(SVM)(T2WI) | 2024 | China and the USA | 0.66 | 0.96 | 0.59 | single center | internal validation | Brain metastases | MRI | NA | 20 | No | No | genetic test reports |
| Wu(in)(ROS3)(SVM)(T2WI) | 2024 | China and the USA | 0.66 | 0.96 | 0.56 | single center | internal validation | Brain metastases | MRI | NA | 20 | No | No | genetic test reports |
| Wu(in)(SMOTE)(SVM)(T2WI) | 2024 | China and the USA | 0.56 | 0.93 | 0.61 | single center | internal validation | Brain metastases | MRI | NA | 20 | No | No | genetic test reports |
| Wu(in)(ADASYN)(SVM)(T2WI) | 2024 | China and the USA | 0.63 | 0.94 | 0.68 | single center | internal validation | Brain metastases | MRI | NA | 20 | No | No | genetic test reports |
| Wu(in)(bSMOTE)(SVM)(T2WI) | 2024 | China and the USA | 0.58 | 0.93 | 0.64 | single center | internal validation | Brain metastases | MRI | NA | 20 | No | No | genetic test reports |
| Wu(in)(SVMSMOTE)(SVM)(T2WI) | 2024 | China and the USA | 0.55 | 0.96 | 0.61 | single center | internal validation | Brain metastases | MRI | NA | 20 | No | No | genetic test reports |
| Wu(in)(cc)(SVM)(T2WI) | 2024 | China and the USA | 0.50 | 0.90 | 0.52 | single center | internal validation | Brain metastases | MRI | NA | 20 | No | No | genetic test reports |
| Wu(in)(RUS)(SVM)(T2WI) | 2024 | China and the USA | 0.48 | 0.90 | 0.54 | single center | internal validation | Brain metastases | MRI | NA | 20 | No | No | genetic test reports |
| Wu(in)(TL)(SVM)(T2WI) | 2024 | China and the USA | 0.50 | 0.88 | 0.52 | single center | internal validation | Brain metastases | MRI | NA | 20 | No | No | genetic test reports |
| Wu(in)(NearMiss)(SVM)(T2WI) | 2024 | China and the USA | 0.77 | 0.97 | 0.87 | single center | internal validation | Brain metastases | MRI | NA | 20 | No | No | genetic test reports |
| Wu(in)(SMOTEENN)(SVM)(T2WI) | 2024 | China and the USA | 0.65 | 0.95 | 0.75 | single center | internal validation | Brain metastases | MRI | NA | 20 | No | No | genetic test reports |
| Wu(in)(SMOTEETL)(SVM)(T2WI) | 2024 | China and the USA | 0.57 | 0.93 | 0.66 | single center | internal validation | Brain metastases | MRI | NA | 20 | No | No | genetic test reports |
| Wu(in)(ROS)(RF)(T2WI) | 2024 | China and the USA | 0.94 | 0.99 | 0.98 | single center | internal validation | Brain metastases | MRI | NA | 20 | No | No | genetic test reports |
| Wu(in)(ROS1)(RF)(T2WI) | 2024 | China and the USA | 0.89 | 0.98 | 0.90 | single center | internal validation | Brain metastases | MRI | NA | 20 | No | No | genetic test reports |
| Wu(in)(ROS3)(RF)(T2WI) | 2024 | China and the USA | 0.91 | 0.99 | 0.91 | single center | internal validation | Brain metastases | MRI | NA | 20 | No | No | genetic test reports |
| Wu(in)(SMOTE)(RF)(T2WI) | 2024 | China and the USA | 0.85 | 0.98 | 0.91 | single center | internal validation | Brain metastases | MRI | NA | 20 | No | No | genetic test reports |
| Wu(in)(ADASYN)(RF)(T2WI) | 2024 | China and the USA | 0.86 | 0.98 | 0.92 | single center | internal validation | Brain metastases | MRI | NA | 20 | No | No | genetic test reports |
| Wu(in)(bSMOTE)(RF)(T2WI) | 2024 | China and the USA | 0.87 | 0.98 | 0.93 | single center | internal validation | Brain metastases | MRI | NA | 20 | No | No | genetic test reports |
| Wu(in)(SVMSMOTE)(RF)(T2WI) | 2024 | China and the USA | 0.86 | 0.98 | 0.89 | single center | internal validation | Brain metastases | MRI | NA | 20 | No | No | genetic test reports |
| Wu(in)(cc)(RF)(T2WI) | 2024 | China and the USA | 0.52 | 0.92 | 0.46 | single center | internal validation | Brain metastases | MRI | NA | 20 | No | No | genetic test reports |
| Wu(in)(RUS)(RF)(T2WI) | 2024 | China and the USA | 0.55 | 0.93 | 0.48 | single center | internal validation | Brain metastases | MRI | NA | 20 | No | No | genetic test reports |
| Wu(in)(TL)(RF)(T2WI) | 2024 | China and the USA | 0.56 | 0.94 | 0.70 | single center | internal validation | Brain metastases | MRI | NA | 20 | No | No | genetic test reports |
| Wu(in)(NearMiss)(RF)(T2WI) | 2024 | China and the USA | 0.83 | 0.98 | 0.85 | single center | internal validation | Brain metastases | MRI | NA | 20 | No | No | genetic test reports |
| Wu(in)(SMOTEENN)(RF)(T2WI) | 2024 | China and the USA | 0.91 | 0.99 | 0.97 | single center | internal validation | Brain metastases | MRI | NA | 20 | No | No | genetic test reports |
| Wu(in)(SMOTEETL)(RF)(T2WI) | 2024 | China and the USA | 0.87 | 0.98 | 0.93 | single center | internal validation | Brain metastases | MRI | NA | 20 | No | No | genetic test reports |
| Wu(in)(ROS)(XGBoost)(T2WI) | 2024 | China and the USA | 0.93 | 0.99 | 0.97 | single center | internal validation | Brain metastases | MRI | NA | 20 | No | No | genetic test reports |
| Wu(in)(ROS1)(XGBoost)(T2WI) | 2024 | China and the USA | 0.87 | 0.98 | 0.87 | single center | internal validation | Brain metastases | MRI | NA | 20 | No | No | genetic test reports |
| Wu(in)(ROS3)(XGBoost)(T2WI) | 2024 | China and the USA | 0.90 | 0.99 | 0.91 | single center | internal validation | Brain metastases | MRI | NA | 20 | No | No | genetic test reports |
| Wu(in)(SMOTE)(XGBoost)(T2WI) | 2024 | China and the USA | 0.84 | 0.98 | 0.89 | single center | internal validation | Brain metastases | MRI | NA | 20 | No | No | genetic test reports |
| Wu(in)(ADASYN)(XGBoost)(T2WI) | 2024 | China and the USA | 0.83 | 0.97 | 0.88 | single center | internal validation | Brain metastases | MRI | NA | 20 | No | No | genetic test reports |
| Wu(in)(bSMOTE)(XGBoost)(T2WI) | 2024 | China and the USA | 0.85 | 0.98 | 0.90 | single center | internal validation | Brain metastases | MRI | NA | 20 | No | No | genetic test reports |
| Wu(in)(SVMSMOTE)(XGBoost)(T2WI) | 2024 | China and the USA | 0.83 | 0.97 | 0.85 | single center | internal validation | Brain metastases | MRI | NA | 20 | No | No | genetic test reports |
| Wu(in)(cc)(XGBoost)(T2WI) | 2024 | China and the USA | 0.62 | 0.94 | 0.54 | single center | internal validation | Brain metastases | MRI | NA | 20 | No | No | genetic test reports |
| Wu(in)(RUS)(XGBoost)(T2WI) | 2024 | China and the USA | 0.63 | 0.94 | 0.61 | single center | internal validation | Brain metastases | MRI | NA | 20 | No | No | genetic test reports |
| Wu(in)(TL)(XGBoost)(T2WI) | 2024 | China and the USA | 0.59 | 0.96 | 0.62 | single center | internal validation | Brain metastases | MRI | NA | 20 | No | No | genetic test reports |
| Wu(in)(NearMiss)(XGBoost)(T2WI) | 2024 | China and the USA | 0.84 | 0.98 | 0.87 | single center | internal validation | Brain metastases | MRI | NA | 20 | No | No | genetic test reports |
| Wu(in)(SMOTEENN)(XGBoost)(T2WI) | 2024 | China and the USA | 0.91 | 0.99 | 0.95 | single center | internal validation | Brain metastases | MRI | NA | 20 | No | No | genetic test reports |
| Wu(in)(SMOTEETL)(XGBoost)(T2WI) | 2024 | China and the USA | 0.86 | 0.98 | 0.90 | single center | internal validation | Brain metastases | MRI | NA | 20 | No | No | genetic test reports |
| Xiong(LR)(Clinic) | 2024 | China | 0.79 | 0.53 | 0.80 | single center | internal validation | Lung and mediastinum | CT | ITK-SNAP | 19 | Yes | No | pathological biopsy |
| Xiong(LR)(Rad) | 2024 | China | 0.42 | 0.77 | 0.72 | single center | internal validation | Lung and mediastinum | CT | ITK-SNAP | 19 | No | No | pathological biopsy |
| Xiong(LR)(Com) | 2024 | China | 0.63 | 0.82 | 0.87 | single center | internal validation | Lung and mediastinum | CT | ITK-SNAP | 19 | Yes | No | pathological biopsy |
| Xiong(SVM)(Clinic) | 2024 | China | 0.68 | 0.88 | 0.81 | single center | internal validation | Lung and mediastinum | CT | ITK-SNAP | 19 | Yes | No | pathological biopsy |
| Xiong(SVM)(Rad) | 2024 | China | 0.63 | 0.82 | 0.73 | single center | internal validation | Lung and mediastinum | CT | ITK-SNAP | 19 | No | No | pathological biopsy |
| Xiong(SVM)(Com) | 2024 | China | 0.90 | 0.59 | 0.87 | single center | internal validation | Lung and mediastinum | CT | ITK-SNAP | 19 | Yes | No | pathological biopsy |
| Xiong(RF)(Clinic) | 2024 | China | 0.68 | 0.77 | 0.78 | single center | internal validation | Lung and mediastinum | CT | ITK-SNAP | 19 | Yes | No | pathological biopsy |
| Xiong(RF)(Rad) | 2024 | China | 0.63 | 0.47 | 0.60 | single center | internal validation | Lung and mediastinum | CT | ITK-SNAP | 19 | No | No | pathological biopsy |
| Xiong(RF)(Com) | 2024 | China | 0.84 | 0.65 | 0.85 | single center | internal validation | Lung and mediastinum | CT | ITK-SNAP | 19 | Yes | No | pathological biopsy |
| Xiong(LR)(Rad) | 2025 | China | 0.83 | 0.71 | 0.81 | single center | internal validation | Lung and mediastinum | CT | ITK-SNAP | 19 | No | Yes | pathological biopsy |
| Xiong(LR)(Clinic) | 2025 | China | 0.78 | 0.76 | 0.81 | single center | internal validation | Lung and mediastinum | CT | ITK-SNAP | 19 | Yes | Yes | pathological biopsy |
| Xiong(LR)(Com) | 2025 | China | 0.89 | 0.82 | 0.90 | single center | internal validation | Lung and mediastinum | CT | ITK-SNAP | 19 | Yes | Yes | pathological biopsy |
| Zhang(ex)(Clinic) | 2025 | China | 0.71 | 0.98 | 0.75 | multi-center | external validation | Lung and mediastinum | CT | 3D Slicer | 22 | Yes | Yes | genetic test reports |
| Zhang(ex)(2D Rad) | 2025 | China | 0.68 | 0.98 | 0.77 | multi-center | external validation | Lung and mediastinum | CT | 3D Slicer | 22 | No | Yes | genetic test reports |
| Zhang(ex)(3D Rad) | 2025 | China | 0.75 | 0.98 | 0.82 | multi-center | external validation | Lung and mediastinum | CT | 3D Slicer | 22 | No | Yes | genetic test reports |
| Zhang(ex)(P-Rad) | 2025 | China | 0.77 | 0.98 | 0.77 | multi-center | external validation | Lung and mediastinum | CT | 3D Slicer | 22 | No | Yes | genetic test reports |
| Zhang(ex)(Com) | 2025 | China | 0.83 | 0.99 | 0.90 | multi-center | external validation | Lung and mediastinum | CT | 3D Slicer | 22 | Yes | Yes | genetic test reports |

Abbreviations: NA, not available.
