# Supplementary material for: Predicting T790M mutation status in non-small cell lung cancer based on radiomics: A systematic review and meta-analysis
Source: PLoS One. 2026 Jul 8;21(7):e0353257. doi: 10.1371/journal.pone.0353257 (PMC13345267; doi:10.1371/journal.pone.0353257)
Supplement: S1 File — (DOCX) [file pone.0353257.s005.docx]

**Supplementary file. The review protocol.**

Title: Predicting T790M mutation status in non-small cell lung cancer based on radiomics: a systematic review and meta-analysis

Question: In recent years, epidermal growth factor receptor tyrosine kinase inhibitors (EGFR-TKIs) have transformed outcomes for patients with EGFR-mutated lung cancer. In the National Comprehensive Cancer Network (NCCN) guidelines, many first- or second-generation EGFR-TKIs are recommended for first-line treatment. However, unfortunately, after approximately 9 to 14 months, resistance inevitably develops. T790 M is the primary cause of acquired resistance, with 60% of patients developing T790 M after initial response to first-line EGFR-TKI therapy. In clinical practice, early detection of the presence of T790 M mutations is equally important during disease diagnosis or disease progression. Radiomics is a non-invasive method for assessing the entire lesion, reflecting the tumor microenvironment, and thereby predicting prognosis. Several studies have shown that machine learning models constructed using features extracted from lung cancer patients via MRI, CT, PET-CT, and other methods can distinguish NSCLC patients with T790M resistance. However, the lack of standardized radiomics workflows limits the robustness and reproducibility of these models.

This study aims to systematically review and comprehensively summarize the application of radiomics in the early identification of T790M gene mutations in non-small cell lung cancer, with a focus on diagnostic performance, sensitivity, and specificity, providing potential reference tools for clinicians to assess T790M status and improve the accuracy of early diagnosis.

**18. * Condition or domain being studied.**

Early identification of the T790M drug resistance gene in non-small cell lung cancer.

**19. * Participants/population.**

People who were diagnosed with lung cancer.

**20. * Intervention(s), exposure(s).**

The observation group includes patients with non-small cell lung cancer harboring the T790M mutation. Studies that do not strictly exclude other mutated genes in the T790M mutation analysis will be excluded.

**21. * Comparator(s)/control.**

Histopathologic results were used as the reference standard to compare the performance of radiomics models.

**22. * Types of study to be included**

Only nonrandomized study types will be included.

Included Observational studies

Excluded non-English publications, case reports, editorials, conference abstracts, review articles

**24. * Main outcome(s).**

The main outcome measures encompassed sensitivity, specificity, positive likelihood ratio, negative likelihood ratio, and the area under the curve

Measures of effect

sensitivity, specificity, accuracy, and the area under the receiver operating characteristic curve (AUC).

1. *** Additional outcome(s).**

NA

**26. * Data extraction (selection and coding).**

Two independent reviewers extracted data from the included cohort study. The extracted information included: (1) general data: authors’ names, year of publication, country, and sample size; (2) participant characteristics: age, sex, diagnosis, and previous treatment regimen(if any); (3) intervention and control group details:imaging modality; and (4) outcomes: diagnostic performance metrics (Accuracy, sensitivity, specificity, AUC). Any discrepancies in data extraction were resolved through discussion and consensus between the reviewers.

**27. * Risk of bias (quality) assessment.**

Use the Radiomics Quality Score (RQS) checklist and the modified Quality Assessment of Diagnostic Accuracy Studies (QUADAS-2) tool to assess the included articles.

**28. * Strategy for data synthesis.**

1.Statistical analysis was performed using STATA 14.0 (StataCorp LLC, College Station, Texas, USA). A summary receiver operating characteristic (ROC) curve was created by analyzing 2 × 2 table data, with AUC as the measure of diagnostic accuracy.

2.The inconsistency index (I²) statistic, a kind of index to represent the percent of diversity that is due to heterogeneity rather than chance, was applied to quantify the magnitude of heterogeneity derived from the random-effects Mantel-Haenszel model. It indicated the significant heterogeneity if the I² is greater than 50%.

3.Begg’s funnel plots and Egger regression asymmetry tests was operated to evaluate the probability of publication bias at a significance level of 10%. The trim-and-fill method was utilized to estimate the number of theoretically missing studies. Sensitivity analysis was performed to test the reliability of the total pooled results by omitting the study sequentially.

**29. * Analysis of subgroups or subsets.**

In subgroup analyses, specific covariates were examined to determine the source of heterogeneity. Study setting (multicenter or single-center), study design (prospective or retrospective), presence of an external validation cohort, localization, segmentation method and dimension, CT scan, MRI, or PET-CT-based model, type of classifier used, and validation method (cross-validation or validation cohort) were considered for evaluation.

Identifier: CRD420251130164
