# Supplementary material for: Predicting T790M mutation status in non-small cell lung cancer based on radiomics: A systematic review and meta-analysis
Source: PLoS One. 2026 Jul 8;21(7):e0353257. doi: 10.1371/journal.pone.0353257 (PMC13345267; doi:10.1371/journal.pone.0353257)
Supplement: Attachment — Submitted filename: Rebuttal letter.docx [file pone.0353257.s006.docx]

**Rebuttal Letter**

Dear Promila Bishnoi:

First and foremost, l would like to extend my sincere gratitude for the time and effort you have dedicated to reviewing my manuscript. Your invaluable feedback greatly aids in enhancing the quality of my research, Now, every comment was meticulously addressed, ensuring that the necessary adjustments were made to enhance the paper's rigor and clarity. l believe that these revisions have significantly improved the manuscript, rendering it more comprehensive and refined.

Herein, l provide detailed responses to the comments made by the reviewers:

**Reviewer's comments:**

In the case of your submission, we note that language restrictions are imposed on the literature searches without adequate justification. There is increasing recognition that exclusion of studies published in other languages can increase the risk of bias in systematic reviews. We feel that any restrictions should be justified based on scientific reasons rather than resource constraints. As a result, we have concerns about the comprehensiveness of the review and the validity of the conclusions.

***Response:****We have expanded our database and re-retrieved non-English articles to reduce language bias. Through an additional retrieval process, we re-added two studies and modified each section of the data individually. Concurrently, we updated the ROS data quality assessment tool and evaluated the radiomics workflow using the latest version, RQS 2.0.*

The invaluable revisions have been made with respect to the feedback received, and l hope they meet your expectations. Once again, thank you for your constructive critique.

In conclusion, l hope the revised manuscript will be deemed suitable for publication. l eagerly await your further comments or decision.

Sincerely,

Hongyang Chen

E-mail:3497258059@qq.com

Tel:18801230669
