# Supplementary material for: Predicting T790M mutation status in non-small cell lung cancer based on radiomics: A systematic review and meta-analysis
Source: PLoS One. 2026 Jul 8;21(7):e0353257. doi: 10.1371/journal.pone.0353257 (PMC13345267; doi:10.1371/journal.pone.0353257)
Supplement: Attachment — Submitted filename: Response to Reviewers.docx [file pone.0353257.s008.docx]

**Response to Reviewer Comments**

*Response to the review of the paper entitled: “****Predicting T790M mutation status in non-small cell lung cancer based on radiomics: a systematic review and meta-analysis****”*

February 8, 2026

Hongyang Chen

We acknowledge and sincerely appreciate the review by the two reviewers. We have revised the manuscript accordingly. In this document, we provide our responses. All the changes are highlighted in red color in the revised manuscript.

**Reviewer(s)’ Comments to Author:**

**Reviewer 1**
1. All included studies were conducted in China, with no representation from other geographic regions or healthcare systems. This raises serious concerns regarding generalizability, given known differences in EGFR mutation epidemiology across ethnic populations, imaging acquisition protocols and scanner characteristics, and clinical workflows and reference-standard practices.

***Response*:** Thank you for your valuable commentary. First, we wish to clarify that the concentration of the study's geographic focus did not stem from subjective selection or search bias. To maximize the collection of relevant global literature, we implemented a systematic and comprehensive search strategy. Second, we fully concur with your perspective and have therefore expanded the discussion on geographical limitations within the manuscript. Please see page 25, lines 508-521 for details, and all the changes are highlighted in red color.
2.The meta-analysis pools studies predicting T790M mutation status using radiomics derived from primary lung tumors, brain metastases, and spinal metastases. These represent biologically and radiologically distinct disease contexts, with different tumor microenvironments, imaging contrasts, and mechanisms potentially related to T790M emergence. Pooling such heterogeneous targets under a single diagnostic estimate is problematic and undermines biological interpretability. At minimum, these entities should be analyzed separately, or the pooled results should be clearly framed as exploratory rather than definitive.

***Response*:** Thank you for your helpful feedback. We fully agree that primary tumors and different metastatic sites (such as brain and spine) exhibit fundamental biological and radiological differences. Combining them in analyses may obscure specific signals and weaken the interpretability of results. Therefore, we conducted separate subgroup meta-analyses by tumor location (primary tumor, brain metastases, other metastases). Additionally, we explicitly defined this as an “exploratory” estimation in the methodology section to emphasize its heterogeneity. Please see page 10, lines 219-221, and page 17, lines 329-334 for details, and all the changes are highlighted in red color.

3.When multiple models were reported within a single study, the authors extracted only the model with the highest AUC for meta-analysis. This introduces a clear optimism bias and artificially inflates pooled performance estimates. A meta-analysis should reflect typical model performance, not selectively chosen best-case scenarios. This methodological choice significantly compromises the credibility of the reported pooled AUC, sensitivity, and specificity.

***Response*:** Thanks for your considerate suggestion. We re-added all datasets from internal and external validation studies, yielding a total of 165 datasets. After hierarchical pooling of the studies, the AUC for internal validation datasets was 0.91 (95% CI: 0.88-0.93), while the AUC for external validation datasets was 0.81 (95% CI: 0.77-0.84). The results demonstrate the robustness and strong diagnostic capability of the predictive model. Please see Page 16 lines 296-307.

4. All included studies are retrospective, and many rely exclusively on internal validation or cross-validation strategies. External validation, when present, is limited and heterogeneous. Notably, none of the studies achieved RQS credit for prospective validation, clinical utility assessment, or deployment readiness. This directly contradicts the manuscript’s repeated references to “clinical application prospects.”

***Response*:** Thank you so much for your helpful comment. We have revised the wording regarding “clinical application prospects” in the text, emphasizing that the conclusions are based on retrospective data.Furthermore, we highlighted this limitation prominently at the end of the paper. Please see page 31, lines 597-604 for details, and all the changes are highlighted in red color.
5.The subgroup analysis reports that studies with higher RQS scores (>20) demonstrate lower sensitivity and specificity. This observation is presented descriptively without adequate interpretation. RQS is designed to assess methodological rigor, not performance optimization. Lower performance in higher-RQS studies is more plausibly explained by reduced overfitting and more conservative validation. The manuscript fails to acknowledge this, risking misleading interpretation.

***Response*:** Thank you for your kind suggestions. We fully agree with the reviewers' perspective that the Radiomics Quality Score (RQS) evaluates methodological rigor rather than performance optimization, and that more conservative validation in studies with high RQS may explain the observed performance trends. We have incorporated a relevant description into the Discussion section as per your suggestion.Please see page 28, lines 526-532. All the changes are highlighted in red color.

6. More references on lung cancer studies should be added to attract a broader readership i.e., PMID: 40339270, PMID: 39930275.

***Response*:** Thank you for your careful advice. We have added these two references to the introduction section. Please see page 5, lines 127-130 for details. All the changes are highlighted in red color.
7. Specificity heterogeneity is high (*I²* >70%), yet the subgroup and meta-regression analyses are based on small numbers of studies per subgroup. Multiple subgroup comparisons are conducted without adjustment for multiple testing. Despite this, the manuscript draws strong conclusions regarding MRI superiority over CT, differences between segmentation software (ITK-SNAP vs 3D Slicer). These findings should be interpreted as hypothesis-generating only, not confirmatory.

***Response*:** Thanks for your detailed work. We have revised the sections involving subgroup comparisons to explicitly state that these analyses are based on exploratory comparisons of small samples or to emphasize the tentative nature of conclusions by incorporating terms such as “may.” Please see page 23, lines 413-433, Please see page 26, lines 489-499 for details, and all the changes are highlighted in red color.
8. The conclusion that MRI outperforms CT is confounded by the fact that MRI studies predominantly involve brain metastases, while CT studies focus on thoracic disease. The analysis does not adequately disentangle modality effects from anatomical site effects, rendering the modality comparison unreliable.

***Response*:** Thanks for your detailed work. We will remove definitive conclusions such as “MRI is superior to CT” from the original text. In the ‘Results’ section, the relevant statement will be revised to read: “In this exploratory analysis, based on limited studies, numerical differences in specificity were reported between MRI models for brain metastases and CT models for thoracic disease. However, due to complete covariation between imaging modalities and anatomical sites, this discrepancy cannot be attributed to the modality itself.” Please see age 23, lines 413-433 for details and all the changes are highlighted in red color.
9. Reference standards for T790M mutation status vary widely, including tissue biopsy, plasma ctDNA, and genetic reports. These methods differ substantially in sensitivity and false-negative rates, yet are treated as equivalent gold standards. This heterogeneity is insufficiently addressed and likely biases pooled diagnostic accuracy estimates.

***Response*:** Thanks for your thoughtful comments. Per your suggestion, we performed a subgroup analysis of different reference standards for the T790M mutation. The implications for diagnostic accuracy are discussed in the discussion section. Please see page 20, lines 346-350, and page 27, lines 506-521. All the changes are highlighted in red color.
10. The Fagan nomogram analysis assumes a pre-test probability of 40%, which is not clearly justified and may not reflect real-world prevalence in many clinical scenarios. Moreover, no comparison is made with established diagnostic strategies (e.g., liquid biopsy), and no decision-curve analysis is provided. As such, claims of meaningful clinical utility are premature.

***Response*:** Thanks for your detailed work.We sincerely apologize for the lack of clarity in the original text regarding the origin of the prior probability. In the revision, we have emphasized in the conclusion on prior probability that it represents the weighted pooled prevalence automatically calculated by the STATA midas command during our diagnostic meta-analysis. This calculation is based on the raw 2x2 contingency table data (true positives, false positives, etc.) from all included studies. This value objectively reflects the average population prevalence of the T790M mutation across the existing literature synthesized in this meta-analysis. Your correction regarding the lack of comparison with existing standards (e.g., liquid biopsy) and decision curve analysis is entirely correct. Our meta-analysis primarily assessed diagnostic accuracy (sensitivity/specificity) and explored its impact on diagnostic reasoning (via predictive values and Fagan diagrams), but did not directly evaluate effects on treatment decisions or patient outcomes—the core of “clinical utility.”We will replace the term “clinical utility” with more precise language, such as “has the potential to guide clinical probability.” Additionally, incorporating your previous review comment, we have strengthened the discussion of reference standard heterogeneity in the Results and Discussion sections. We specifically note that current estimates of model true specificity may be biased due to the lack of prospective, externally validated studies using tissue biopsy as the gold standard, further supporting the necessity for higher-level clinical validation. Furthermore, the Discussion section explicitly states: No head-to-head comparisons were conducted between the radiomics model and existing diagnostic strategies like liquid biopsy, nor were decision curve analyses performed to assess its net benefit in clinical decision-making. This represents a significant limitation of the study. The future clinical value of radiomics is more likely to lie in its role as a non-invasive, reproducible supplementary tool, providing decision-making references for patients unsuitable for standard biopsy. Its precise clinical utility—specifically, whether it can improve final decisions and patient outcomes—needs to be validated in future prospective studies through comparisons with standard strategies and formal health economic or decision impact analyses. Please see page 21, lines 375-388 for details. All the changes are highlighted in red color.
**Independent Review Report, Reviewer 2**
1.Article type. This manuscript is not an original research, but a meta-analysis paper. If allowed, it should be categorized as meta-analysis or review rather than original research.

***Response*:** Thank you so much for your helpful suggestion. We sincerely apologize for the error caused by our oversight. We will immediately contact the journal editorial office to confirm and ensure the manuscript type is correctly marked as “Systematic Review and Meta-Analysis” in the system to avoid any misunderstanding.

2.Abstract. Generally ok.

***Response*:** We appreciate the reviewers' overall approval of the abstract section.

We have simultaneously revised and streamlined the corresponding parts of the abstract based on your valuable comments and those of other reviewers—such as clarifying the study's geographical limitations and adjusting overly emphatic statements regarding clinical efficacy—to ensure it more clearly and rigorously reflects the core findings and positioning of the full text.

We sincerely thank you for your review and affirmation.
3.Introduction. It is very long and should be shortened by about 25% for greater conciseness and readability. A lot of details are contained in the Results section, so here it is preferable to focus as much as possible on the knowledge gaps this study is aimed to fill and how. In this context, please consider the addition of few words (around lines 129-136) emphasizing the potential importance of AI and radiomics in developing imaging biobanks that could be used for fostering personalized patient care (see e.g. doi 10.1007/s00330-021-08431-6 as potential useful reference).

***Response*:** Thank you so much for your helpful advice. We have streamlined the content of the introduction. Additionally, we have added material on the potential significance of artificial intelligence and radiology in developing imaging biobanks that can promote personalized patient care.Please see page 5, lines 124-127 for details. All the changes are highlighted in red color.

4 Results. At lines 366-368, please briefly recall some of the most relevant points from the literature taken into consideration where no significant changes were found. If necessary, the caption of Fig. 5 should be modified accordingly to avoid any redundancies with the revised text.

***Response*:** Thanks for your detailed work. We have revised the results section and figure captions of the manuscript according to your suggestions. Please see page 21, lines 364- 367 for details and all the changes are highlighted in red color.
5. Discussion. It is quite long and should be shortened by about 25%. Moreover, it should focus not only on summarizing the key findings from the literature, but also discussing them to find a plausible explanation to the findings, e.g., why did MRI outperform CT? why was 3DSlicer better as segmentation software? Furthermore, at line 387 please replace the subtitle 'Key finding' with a brief summary of the actual key findings as described in the following text.

***Response*:** Thanks for your thoughtful comments. Per your suggestion, we have removed redundant descriptions and minor details, reducing the discussion section by approximately 20%. Although this falls short of the 25% reduction target, it represents the optimal outcome after balancing the article's overall coherence with the feedback from another reviewer. Secondly, the advantages of imaging equipment and segmentation software have been incorporated into the discussion. Additionally, the heading “key findings” has been replaced with the more precise term “key findings.” Once again, thank you for your meticulous and diligent work, which has significantly enhanced our manuscript. Please see page 22, lines 392-403, pages 23-24, lines 413-450, and page 26, lines 478-501, and all the changes are highlighted in red color.
