# Supplementary material for: Predicting T790M mutation status in non-small cell lung cancer based on radiomics: A systematic review and meta-analysis
Source: PLoS One. 2026 Jul 8;21(7):e0353257. doi: 10.1371/journal.pone.0353257 (PMC13345267; doi:10.1371/journal.pone.0353257)
Supplement: Attachment — Submitted filename: Reviewer comments.docx [file pone.0353257.s007.docx]

**Manuscript Number:** PONE-D-25-64207R1

**Title:** Predicting T790M Mutation Status in Non-Small Cell Lung Cancer Based on Radiomics: A Systematic Review and Meta-analysis

**Reviewer Comments**

The authors present a systematic review and meta-analysis evaluating the diagnostic performance of radiomics models for predicting EGFR T790M mutation status in non-small cell lung cancer (NSCLC). The topic is clinically relevant, particularly considering the growing importance of non-invasive biomarkers and AI-driven imaging approaches in precision oncology. The manuscript is generally well organized, and the authors have made substantial revisions in response to previous reviewer comments. The inclusion of subgroup analyses, RQS evaluation, and QUADAS-2 assessment strengthens the methodological framework of the study.

**Minor Comments**

1. The Introduction remains slightly lengthy and could still be condensed for better readability. Some background information regarding EGFR-TKI resistance mechanisms may be streamlined.
2. Several grammatical inconsistencies and typographical issues remain throughout the manuscript. Examples include:
   - “We usd the RQS 2.0 checklist…”
   - “assesse the quality…”
   - “use of CT imaging offers higher sensitivity for fixation…”

A careful professional English-language revision is recommended.

3. Figure legends should provide slightly more methodological detail to improve standalone interpretability.

Once these corrections are adequately addressed, the manuscript may be considered suitable for publication.
