# Supplementary material for: Predicting T790M mutation status in non-small cell lung cancer based on radiomics: A systematic review and meta-analysis
Source: PLoS One. 2026 Jul 8;21(7):e0353257. doi: 10.1371/journal.pone.0353257 (PMC13345267; doi:10.1371/journal.pone.0353257)
Supplement: Attachment — Submitted filename: Response_to_Reviewers_auresp_2.docx [file pone.0353257.s009.docx]

**Response to Reviewer Comments**

*Response to the review of the paper entitled: “****Predicting T790M mutation status in non-small cell lung cancer based on radiomics: a systematic review and meta-analysis****”*

May 27, 2026

Hongyang Chen

We acknowledge and sincerely appreciate the review. We have revised the manuscript accordingly. In this document, we provide our responses. All the changes are highlighted in red color in the revised manuscript.

**Reviewer(s)’ Comments to Author:**

1. Careful English language editing is recommended to correct remaining grammatical and typographical errors.

***Response*:** Thank you for your valuable commentary. The manuscript has undergone careful English language editing. All grammatical and typographical errors have been corrected, and the clarity of the text has been improved throughout. The changes are highlighted in red color.
2.The subgroup findings should be interpreted more cautiously as exploratory observations.

***Response*:** Thank you for your helpful feedback. We have already emphasized the exploratory nature of subgroup analyses in the “Limitations” section. Please see page 31, lines 595-603, and all the changes are highlighted in red color.

3.Methodological explanations and figure legends could be slightly clearer for better readability.

***Response*:** Thanks for your considerate suggestion. We have carefully revised the methodology and legends to improve readability. Please see Page 8, lines 183-185，Page 17, lines 312-315, and Page 21, line 371.
