# Supplementary material for: Predicting T790M mutation status in non-small cell lung cancer based on radiomics: A systematic review and meta-analysis
Source: PLoS One. 2026 Jul 8;21(7):e0353257. doi: 10.1371/journal.pone.0353257 (PMC13345267; doi:10.1371/journal.pone.0353257)
Supplement: Peer review history

# Author comment: Author response to Decision Letter 0

**DOI:** 10.1371/journal.pone.0353257.r001

2 Dec 2025

Attachment Submitted filename: Rebuttal letter.docx

# Peer review: Decision Letter 0

**DOI:** 10.1371/journal.pone.0353257.r002

**Authors:** Hamidreza Montazeri Aliabadi

28 Jan 2026

PONE-D-25-64207

Predicting T790M mutation status in non-small cell lung cancer based on radiomics: a systematic review and meta-analysis

PLOS One

Dear Dr. Chen,

Thank you for submitting your manuscript to PLOS ONE. After careful consideration, we feel that it has merit but does not fully meet PLOS ONE’s publication criteria as it currently stands. Therefore, we invite you to submit a revised version of the manuscript that addresses the points raised during the review process.

Please submit your revised manuscript by Mar 13 2026 11:59PM. If you will need more time than this to complete your revisions, please reply to this message or contact the journal office at plosone@plos.org. When you're ready to submit your revision, log on to https://www.editorialmanager.com/pone/ and select the 'Submissions Needing Revision' folder to locate your manuscript file.

Please include the following items when submitting your revised manuscript:

- A letter that responds to each point raised by the academic editor and reviewer(s). You should upload this letter as a separate file labeled 'Response to Reviewers'.
- A marked-up copy of your manuscript that highlights changes made to the original version. You should upload this as a separate file labeled 'Revised Manuscript with Track Changes'.
- An unmarked version of your revised paper without tracked changes. You should upload this as a separate file labeled 'Manuscript'.

If you would like to make changes to your financial disclosure, please include your updated statement in your cover letter. Guidelines for resubmitting your figure files are available below the reviewer comments at the end of this letter.

If applicable, we recommend that you deposit your laboratory protocols in protocols.io to enhance the reproducibility of your results. Protocols.io assigns your protocol its own identifier (DOI) so that it can be cited independently in the future. For instructions see: https://journals.plos.org/plosone/s/submission-guidelines#loc-laboratory-protocols. Additionally, PLOS ONE offers an option for publishing peer-reviewed Lab Protocol articles, which describe protocols hosted on protocols.io. Read more information on sharing protocols at https://plos.org/protocols?utm_medium=editorial-email&utm_source=authorletters&utm_campaign=protocols.

We look forward to receiving your revised manuscript.

Kind regards,

Hamidreza Montazeri Aliabadi

Academic Editor

PLOS One

Journal Requirements:

When submitting your revision, we need you to address these additional requirements.

1. Please ensure that your manuscript meets PLOS ONE's style requirements, including those for file naming. The PLOS ONE style templates can be found at

https://journals.plos.org/plosone/s/file?id=wjVg/PLOSOne_formatting_sample_main_body.pdf and https://journals.plos.org/plosone/s/file?id=ba62/PLOSOne_formatting_sample_title_authors_affiliations.pdf

2. We note that the grant information you provided in the ‘Funding Information’ and ‘Financial Disclosure’ sections do not match.

When you resubmit, please ensure that you provide the correct grant numbers for the awards you received for your study in the ‘Funding Information’ section.

3. Thank you for stating in your Funding Statement:

[This work was supported by the National Key Research and Development Program of China (NO. 2023YFC3503301), High Level Chinese Medical Hospital Promotion Project (NO. HLCMHPP2023085).].

Please provide an amended statement that declares *all* the funding or sources of support (whether external or internal to your organization) received during this study, as detailed online in our guide for authors at http://journals.plos.org/plosone/s/submit-now.  Please also include the statement “There was no additional external funding received for this study.” in your updated Funding Statement.

Please include your amended Funding Statement within your cover letter. We will change the online submission form on your behalf.

4. Thank you for stating the following in your manuscript:

[This work was supported by the National Key Research and Development Program of China (NO. 2023YFC3503301), High Level Chinese Medical Hospital Promotion Project (NO. HLCMHPP2023085).]

We note that you have provided funding information that is currently declared in your Funding Statement. However, funding information should not appear in the Acknowledgments section or other areas of your manuscript. We will only publish funding information present in the Funding Statement section of the online submission form.

Please remove any funding-related text from the manuscript and let us know how you would like to update your Funding Statement. Currently, your Funding Statement reads as follows:

[This work was supported by the National Key Research and Development Program of China (NO. 2023YFC3503301), High Level Chinese Medical Hospital Promotion Project (NO. HLCMHPP2023085).]

Please include your amended statements within your cover letter; we will change the online submission form on your behalf.

5. When completing the data availability statement of the submission form, you indicated that you will make your data available on acceptance. We strongly recommend all authors decide on a data sharing plan before acceptance, as the process can be lengthy and hold up publication timelines. Please note that, though access restrictions are acceptable now, your entire data will need to be made freely accessible if your manuscript is accepted for publication. This policy applies to all data except where public deposition would breach compliance with the protocol approved by your research ethics board. If you are unable to adhere to our open data policy, please kindly revise your statement to explain your reasoning and we will seek the editor's input on an exemption. Please be assured that, once you have provided your new statement, the assessment of your exemption will not hold up the peer review process.

6. Your ethics statement should only appear in the Methods section of your manuscript. If your ethics statement is written in any section besides the Methods, please move it to the Methods section and delete it from any other section. Please ensure that your ethics statement is included in your manuscript, as the ethics statement entered into the online submission form will not be published alongside your manuscript.

7. Please include captions for your Supporting Information files at the end of your manuscript, and update any in-text citations to match accordingly. Please see our Supporting Information guidelines for more information: http://journals.plos.org/plosone/s/supporting-information.

Additional Editor Comments:

We note that the Reviewer comments include recommendations to cite specific previously published works, please review and evaluate these publications to determine whether they are relevant and should be cited. There is no requirement to cite these works unless the editor has indicated otherwise.

[Note: HTML markup is below. Please do not edit.]

Reviewers' comments:

Reviewer's Responses to Questions

**Comments to the Author**

1. Is the manuscript technically sound, and do the data support the conclusions?

The manuscript must describe a technically sound piece of scientific research with data that supports the conclusions. Experiments must have been conducted rigorously, with appropriate controls, replication, and sample sizes. The conclusions must be drawn appropriately based on the data presented.

Reviewer #1: Partly

Reviewer #2: Yes

**********

2. Has the statistical analysis been performed appropriately and rigorously?

Reviewer #1: No

Reviewer #2: Yes

**********

3. Have the authors made all data underlying the findings in their manuscript fully available?

The PLOS Data policy requires authors to make all data underlying the findings described in their manuscript fully available without restriction, with rare exception (please refer to the Data Availability Statement in the manuscript PDF file). The data should be provided as part of the manuscript or its supporting information, or deposited to a public repository. For example, in addition to summary statistics, the data points behind means, medians and variance measures should be available. If there are restrictions on publicly sharing data—e.g. participant privacy or use of data from a third party—those must be specified.

Reviewer #1: No

Reviewer #2: Yes

**********

4. Is the manuscript presented in an intelligible fashion and written in standard English?

PLOS ONE does not copyedit accepted manuscripts, so the language in submitted articles must be clear, correct, and unambiguous. Any typographical or grammatical errors should be corrected at revision, so please note any specific errors here.

Reviewer #1: Yes

Reviewer #2: Yes

**********

5. Review Comments to the Author

Please use the space provided to explain your answers to the questions above. You may also include additional comments for the author, including concerns about dual publication, research ethics, or publication ethics. (Please upload your review as an attachment if it exceeds 20,000 characters)

Reviewer #1: 1. All included studies were conducted in China, with no representation from other geographic regions or healthcare systems. This raises serious concerns regarding generalizability, given known differences in EGFR mutation epidemiology across ethnic populations, imaging acquisition protocols and scanner characteristics, and clinical workflows and reference-standard practices.

2. The meta-analysis pools studies predicting T790M mutation status using radiomics derived from primary lung tumors, brain metastases, and spinal metastases. These represent biologically and radiologically distinct disease contexts, with different tumor microenvironments, imaging contrasts, and mechanisms potentially related to T790M emergence. Pooling such heterogeneous targets under a single diagnostic estimate is problematic and undermines biological interpretability. At minimum, these entities should be analyzed separately, or the pooled results should be clearly framed as exploratory rather than definitive.

3. When multiple models were reported within a single study, the authors extracted only the model with the highest AUC for meta-analysis. This introduces a clear optimism bias and artificially inflates pooled performance estimates. A meta-analysis should reflect typical model performance, not selectively chosen best-case scenarios. This methodological choice significantly compromises the credibility of the reported pooled AUC, sensitivity, and specificity.

4. All included studies are retrospective, and many rely exclusively on internal validation or cross-validation strategies. External validation, when present, is limited and heterogeneous. Notably, none of the studies achieved RQS credit for prospective validation, clinical utility assessment, or deployment readiness. This directly contradicts the manuscript’s repeated references to “clinical application prospects.”

5. The subgroup analysis reports that studies with higher RQS scores (>20) demonstrate lower sensitivity and specificity. This observation is presented descriptively without adequate interpretation. RQS is designed to assess methodological rigor, not performance optimization. Lower performance in higher-RQS studies is more plausibly explained by reduced overfitting and more conservative validation. The manuscript fails to acknowledge this, risking misleading interpretation.

6. More references on lung cancer studies should be added to attract a broader readership i.e., PMID: 40339270, PMID: 39930275.

7. Specificity heterogeneity is high (I² >70%), yet the subgroup and meta-regression analyses are based on small numbers of studies per subgroup. Multiple subgroup comparisons are conducted without adjustment for multiple testing. Despite this, the manuscript draws strong conclusions regarding MRI superiority over CT, differences between segmentation software (ITK-SNAP vs 3D Slicer). These findings should be interpreted as hypothesis-generating only, not confirmatory.

8. The conclusion that MRI outperforms CT is confounded by the fact that MRI studies predominantly involve brain metastases, while CT studies focus on thoracic disease. The analysis does not adequately disentangle modality effects from anatomical site effects, rendering the modality comparison unreliable.

9. Reference standards for T790M mutation status vary widely, including tissue biopsy, plasma ctDNA, and genetic reports. These methods differ substantially in sensitivity and false-negative rates, yet are treated as equivalent gold standards. This heterogeneity is insufficiently addressed and likely biases pooled diagnostic accuracy estimates.

10. The Fagan nomogram analysis assumes a pre-test probability of 40%, which is not clearly justified and may not reflect real-world prevalence in many clinical scenarios. Moreover, no comparison is made with established diagnostic strategies (e.g., liquid biopsy), and no decision-curve analysis is provided. As such, claims of meaningful clinical utility are premature.

Reviewer #2: I have the following comments:

1) Article type. This manuscript is not an original research, but a meta-analysis paper. If allowed, it should be categorized as meta-analysis or review rather than original research.

2) Abstract. Generally ok.

3) Introduction. It is very long and should be shortened by about 25% for greater conciseness and readability. A lot of details are contained in the Results section, so here it is preferable to focus as much as possible on the knowledge gaps this study is aimed to fill and how. In this context, please consider the addition of few words (around lines 129-136) emphasizing the potential importance of AI and radiomics in developing imaging biobanks that could be used for fostering personalized patient care (see e.g. doi 10.1007/s00330-021-08431-6 as potential useful reference).

4) Results. At lines 366-368, please briefly recall some of the most relevant points from the literature taken into consideration where no significant changes were found. If necessary, the caption of Fig. 5 should be modified accordingly to avoid any redundancies with the revised text.

5) Discussion. It is quite long and should be shortened by about 25%. Moreover, it should focus not only on summarizing the key findings from the literature, but also discussing them to find a plausible explanation to the findings, e.g., why did MRI outperform CT? why was 3DSlicer better as segmentation software? Furthermore, at line 387 please replace the subtitle 'Key finding' with a brief summary of the actual key findings as described in the following text.

**********

6. PLOS authors have the option to publish the peer review history of their article (what does this mean?). If published, this will include your full peer review and any attached files.

If you choose “no”, your identity will remain anonymous but your review may still be made public.

**Do you want your identity to be public for this peer review?**  For information about this choice, including consent withdrawal, please see our Privacy Policy.

Reviewer #1: No

Reviewer #2: No

**********

[NOTE: If reviewer comments were submitted as an attachment file, they will be attached to this email and accessible via the submission site. Please log into your account, locate the manuscript record, and check for the action link "View Attachments". If this link does not appear, there are no attachment files.]

To ensure your figures meet our technical requirements, please review our figure guidelines: https://journals.plos.org/plosone/s/figures

You may also use PLOS’s free figure tool, NAAS, to help you prepare publication quality figures: https://journals.plos.org/plosone/s/figures#loc-tools-for-figure-preparation.

NAAS will assess whether your figures meet our technical requirements by comparing each figure against our figure specifications.

# Author comment: Author response to Decision Letter 1

**DOI:** 10.1371/journal.pone.0353257.r003

19 Feb 2026

Response to Reviewer Comments

Response to the review of the paper entitled: “Predicting T790M mutation status in non-small cell lung cancer based on radiomics: a systematic review and meta-analysis”

February 8, 2026

Hongyang Chen

We acknowledge and sincerely appreciate the review by the two reviewers. We have revised the manuscript accordingly. In this document, we provide our responses. All the changes are highlighted in red color in the revised manuscript.

Reviewer(s)’ Comments to Author:

Reviewer 1

1. All included studies were conducted in China, with no representation from other geographic regions or healthcare systems. This raises serious concerns regarding generalizability, given known differences in EGFR mutation epidemiology across ethnic populations, imaging acquisition protocols and scanner characteristics, and clinical workflows and reference-standard practices.

Response: Thank you for your valuable commentary. First, we wish to clarify that the concentration of the study's geographic focus did not stem from subjective selection or search bias. To maximize the collection of relevant global literature, we implemented a systematic and comprehensive search strategy. Second, we fully concur with your perspective and have therefore expanded the discussion on geographical limitations within the manuscript. Please see page 25, lines 508-521 for details, and all the changes are highlighted in red color.

2.The meta-analysis pools studies predicting T790M mutation status using radiomics derived from primary lung tumors, brain metastases, and spinal metastases. These represent biologically and radiologically distinct disease contexts, with different tumor microenvironments, imaging contrasts, and mechanisms potentially related to T790M emergence. Pooling such heterogeneous targets under a single diagnostic estimate is problematic and undermines biological interpretability. At minimum, these entities should be analyzed separately, or the pooled results should be clearly framed as exploratory rather than definitive.

Response: Thank you for your helpful feedback. We fully agree that primary tumors and different metastatic sites (such as brain and spine) exhibit fundamental biological and radiological differences. Combining them in analyses may obscure specific signals and weaken the interpretability of results. Therefore, we conducted separate subgroup meta-analyses by tumor location (primary tumor, brain metastases, other metastases). Additionally, we explicitly defined this as an “exploratory” estimation in the methodology section to emphasize its heterogeneity. Please see page 10, lines 219-221, and page 17, lines 329-334 for details, and all the changes are highlighted in red color.

3.When multiple models were reported within a single study, the authors extracted only the model with the highest AUC for meta-analysis. This introduces a clear optimism bias and artificially inflates pooled performance estimates. A meta-analysis should reflect typical model performance, not selectively chosen best-case scenarios. This methodological choice significantly compromises the credibility of the reported pooled AUC, sensitivity, and specificity.

Response: Thanks for your considerate suggestion. We re-added all datasets from internal and external validation studies, yielding a total of 165 datasets. After hierarchical pooling of the studies, the AUC for internal validation datasets was 0.91 (95% CI: 0.88-0.93), while the AUC for external validation datasets was 0.81 (95% CI: 0.77-0.84). The results demonstrate the robustness and strong diagnostic capability of the predictive model. Please see Page 16 lines 296-307.

4. All included studies are retrospective, and many rely exclusively on internal validation or cross-validation strategies. External validation, when present, is limited and heterogeneous. Notably, none of the studies achieved RQS credit for prospective validation, clinical utility assessment, or deployment readiness. This directly contradicts the manuscript’s repeated references to “clinical application prospects.”

Response: Thank you so much for your helpful comment. We have revised the wording regarding “clinical application prospects” in the text, emphasizing that the conclusions are based on retrospective data.Furthermore, we highlighted this limitation prominently at the end of the paper. Please see page 31, lines 597-604 for details, and all the changes are highlighted in red color.

5.The subgroup analysis reports that studies with higher RQS scores (>20) demonstrate lower sensitivity and specificity. This observation is presented descriptively without adequate interpretation. RQS is designed to assess methodological rigor, not performance optimization. Lower performance in higher-RQS studies is more plausibly explained by reduced overfitting and more conservative validation. The manuscript fails to acknowledge this, risking misleading interpretation.

Response: Thank you for your kind suggestions. We fully agree with the reviewers' perspective that the Radiomics Quality Score (RQS) evaluates methodological rigor rather than performance optimization, and that more conservative validation in studies with high RQS may explain the observed performance trends. We have incorporated a relevant description into the Discussion section as per your suggestion.Please see page 28, lines 526-532. All the changes are highlighted in red color.

6.More references on lung cancer studies should be added to attract a broader readership i.e., PMID: 40339270, PMID: 39930275.

Response: Thank you for your careful advice. We have added these two references to the introduction section. Please see page 5, lines 127-130 for details. All the changes are highlighted in red color.

7. Specificity heterogeneity is high (I² >70%), yet the subgroup and meta-regression analyses are based on small numbers of studies per subgroup. Multiple subgroup comparisons are conducted without adjustment for multiple testing. Despite this, the manuscript draws strong conclusions regarding MRI superiority over CT, differences between segmentation software (ITK-SNAP vs 3D Slicer). These findings should be interpreted as hypothesis-generating only, not confirmatory.

Response: Thanks for your detailed work. We have revised the sections involving subgroup comparisons to explicitly state that these analyses are based on exploratory comparisons of small samples or to emphasize the tentative nature of conclusions by incorporating terms such as “may.” Please see page 23, lines 413-433, Please see page 26, lines 489-499 for details, and all the changes are highlighted in red color.

8. The conclusion that MRI outperforms CT is confounded by the fact that MRI studies predominantly involve brain metastases, while CT studies focus on thoracic disease. The analysis does not adequately disentangle modality effects from anatomical site effects, rendering the modality comparison unreliable.

Response: Thanks for your detailed work. We will remove definitive conclusions such as “MRI is superior to CT” from the original text. In the ‘Results’ section, the relevant statement will be revised to read: “In this exploratory analysis, based on limited studies, numerical differences in specificity were reported between MRI models for brain metastases and CT models for thoracic disease. However, due to complete covariation between imaging modalities and anatomical sites, this discrepancy cannot be attributed to the modality itself.” Please see age 23, lines 413-433 for details and all the changes are highlighted in red color.

9. Reference standards for T790M mutation status vary widely, including tissue biopsy, plasma ctDNA, and genetic reports. These methods differ substantially in sensitivity and false-negative rates, yet are treated as equivalent gold standards. This heterogeneity is insufficiently addressed and likely biases pooled diagnostic accuracy estimates.

Response: Thanks for your thoughtful comments. Per your suggestion, we performed a subgroup analysis of different reference standards for the T790M mutation. The implications for diagnostic accuracy are discussed in the discussion section. Please see page 20, lines 346-350, and page 27, lines 506-521. All the changes are highlighted in red color.

10. The Fagan nomogram analysis assumes a pre-test probability of 40%, which is not clearly justified and may not reflect real-world prevalence in many clinical scenarios. Moreover, no comparison is made with established diagnostic strategies (e.g., liquid biopsy), and no decision-curve analysis is provided. As such, claims of meaningful clinical utility are premature.

Response: Thanks for your detailed work.We sincerely apologize for the lack of clarity in the original text regarding the origin of the prior probability. In the revision, we have emphasized in the conclusion on prior probability that it represents the weighted pooled prevalence automatically calculated by the STATA midas command during our diagnostic meta-analysis. This calculation is based on the raw 2x2 contingency table data (true positives, false positives, etc.) from all included studies. This value objectively reflects the average population prevalence of the T790M mutation across the existing literature synthesized in this meta-analysis. Your correction regarding the lack of comparison with existing standards (e.g., liquid biopsy) and decision curve analysis is entirely correct. Our meta-analysis primarily assessed diagnostic accuracy (sensitivity/specificity) and explored its impact on diagnostic reasoning (via predictive values and Fagan diagrams), but did not directly evaluate effects on treatment decisions or patient outcomes—the core of “clinical utility.”We will replace the term “clinical utility” with more precise language, such as “has the potential to guide clinical probability.” Additionally, incorporating your previous review comment, we have strengthened the discussion of reference standard heterogeneity in the Results and Discussion sections. We specifically note that current estimates of model true specificity may be biased due to the lack of prospective, externally validated studies using tissue biopsy as the gold standard, further supporting the necessity for higher-level clinical validation. Furthermore, the Discussion section explicitly states: No head-to-head comparisons were conducted between the radiomics model and existing diagnostic strategies like liquid biopsy, nor were decision curve analyses performed to assess its net benefit in clinical decision-making. This represents a significant limitation of the study. The future clinical value of radiomics is more likely to lie in its role as a non-invasive, reproducible supplementary tool, providing decision-making references for patients unsuitable for standard biopsy. Its precise clinical utility—specifically, whether it can improve final decisions and patient outcomes—needs to be validated in future prospective studies through comparisons with standard strategies and formal health economic or decision impact analyses. Please see page 21, lines 375-388 for details. All the changes are highlighted in red color.

Independent Review Report, Reviewer 2

1.Article type. This manuscript is not an original research, but a meta-analysis paper. If allowed, it should be categorized as meta-analysis or review rather than original research.

Response: Thank you so much for your helpful suggestion. We sincerely apologize for the error caused by our oversight. We will immediately contact the journal editorial office to confirm and ensure the manuscript type is correctly marked as “Systematic Review and Meta-Analysis” in the system to avoid any misunderstanding.

2.Abstract. Generally ok.

Response: We appreciate the reviewers' overall approval of the abstract section.

We have simultaneously revised and streamlined the corresponding parts of the abstract based on your valuable comments and those of other reviewers—such as clarifying the study's geographical limitations and adjusting overly emphatic statements regarding clinical efficacy—to ensure it more clearly and rigorously reflects the core findings and positioning of the full text.

We sincerely thank you for your review and affirmation.

3.Introduction. It is very long and should be shortened by about 25% for greater conciseness and readability. A lot of details are contained in the Results section, so here it is preferable to focus as much as possible on the knowledge gaps this study is aimed to fill and how. In this context, please consider the addition of few words (around lines 129-136) emphasizing the potential importance of AI and radiomics in developing imaging biobanks that could be used for fostering personalized patient care (see e.g. doi 10.1007/s00330-021-08431-6 as potential useful reference).

Response: Thank you so much for your helpful advice. We have streamlined the content of the introduction. Additionally, we have added material on the potential significance of artificial intelligence and radiology in developing imaging biobanks that can promote personalized patient care.Please see page 5, lines 124-127 for details. All the changes are highlighted in red color.

4 Results. At lines 366-368, please briefly recall some of the most relevant points from the literature taken into consideration where no significant changes were found. If necessary, the caption of Fig. 5 should be modified accordingly to avoid any redundancies with the revised text.

Response: Thanks for your detailed work. We have revised the results section and figure captions of the manuscript according to your suggestions. Please see page 21, lines 364- 367 for details and all the changes are highlighted in red color.

5. Discussion. It is quite long and should be shortened by about 25%. Moreover, it should focus not only on summarizing the key findings from the literature, but also discussing them to find a plausible explanation to the findings, e.g., why did MRI outperform CT? why was 3DSlicer better as segmentation software? Furthermore, at line 387 please replace the subtitle 'Key finding' with a brief summary of the actual key findings as described in the following text.

Response: Thanks for your thoughtful comments. Per your suggestion, we have removed redundant descriptions and minor details, reducing the discussion section by approximately 20%. Although this falls short of the 25% reduction target, it represents the optimal outcome after balancing the article's overall coherence with the feedback from another reviewer. Secondly, the advantages of imaging equipment and segmentation software have been incorporated into the discussion. Additionally, the heading “key findings” has been replaced with the more precise term “key findings.” Once again, thank you for your meticulous and diligent work, which has significantly enhanced our manuscript. Please see page 22, lines 392-403, pages 23-24, lines 413-450, and page 26, lines 478-501, and all the changes are highlighted in red color.

Attachment Submitted filename: Response to Reviewers.docx

# Peer review: Decision Letter 1

**DOI:** 10.1371/journal.pone.0353257.r004

**Authors:** Hamidreza Montazeri Aliabadi, Bardia Rodd

27 May 2026

PONE-D-25-64207R1

Predicting T790M mutation status in non-small cell lung cancer based on radiomics: a systematic review and meta-analysis

PLOS One

Dear Dr. Chen,

Thank you for submitting your manuscript to PLOS ONE. After careful consideration, we feel that it has merit but does not fully meet PLOS ONE’s publication criteria as it currently stands. Therefore, we invite you to submit a revised version of the manuscript that addresses the points raised during the review process.

Please submit your revised manuscript by Jul 11 2026 11:59PM. If you will need more time than this to complete your revisions, please reply to this message or contact the journal office at plosone@plos.org. When you're ready to submit your revision, log on to https://www.editorialmanager.com/pone/ and select the 'Submissions Needing Revision' folder to locate your manuscript file.

Please include the following items when submitting your revised manuscript:

- A letter that responds to each point raised by the academic editor and reviewer(s). You should upload this letter as a separate file labeled 'Response to Reviewers'.
- A marked-up copy of your manuscript that highlights changes made to the original version. You should upload this as a separate file labeled 'Revised Manuscript with Track Changes'.
- An unmarked version of your revised paper without tracked changes. You should upload this as a separate file labeled 'Manuscript'.

If you would like to make changes to your financial disclosure, please include your updated statement in your cover letter. Guidelines for resubmitting your figure files are available below the reviewer comments at the end of this letter.

If applicable, we recommend that you deposit your laboratory protocols in protocols.io to enhance the reproducibility of your results. Protocols.io assigns your protocol its own identifier (DOI) so that it can be cited independently in the future. For instructions see: https://journals.plos.org/plosone/s/submission-guidelines#loc-laboratory-protocols. Additionally, PLOS ONE offers an option for publishing peer-reviewed Lab Protocol articles, which describe protocols hosted on protocols.io. Read more information on sharing protocols at https://plos.org/protocols?utm_medium=editorial-email&utm_source=authorletters&utm_campaign=protocols.

As the corresponding author, your ORCID iD is verified in the submission system and will appear in the published article. PLOS supports the use of ORCID, and we encourage all coauthors to register for an ORCID iD and use it as well. Please encourage your coauthors to verify their ORCID iD within the submission system before final acceptance, as unverified ORCID iDs will not appear in the published article. *Only*  the individual author can complete the verification step; PLOS staff *cannot*  verify ORCID iDs on behalf of authors.

We look forward to receiving your revised manuscript.

Kind regards,

Bardia Rodd, Ph.D.

Academic Editor

PLOS One

Journal Requirements:

1. If the reviewer comments include a recommendation to cite specific previously published works, please review and evaluate these publications to determine whether they are relevant and should be cited. There is no requirement to cite these works unless the editor has indicated otherwise.

2. Please review your reference list to ensure that it is complete and correct. If you have cited papers that have been retracted, please include the rationale for doing so in the manuscript text, or remove these references and replace them with relevant current references. Any changes to the reference list should be mentioned in the rebuttal letter that accompanies your revised manuscript. If you need to cite a retracted article, indicate the article’s retracted status in the References list and also include a citation and full reference for the retraction notice.

Additional Editor Comments:

There are very minor items left to revised.

[Note: HTML markup is below. Please do not edit.]

Reviewer's Responses to Questions

**Comments to the Author**

1. If the authors have adequately addressed your comments raised in a previous round of review and you feel that this manuscript is now acceptable for publication, you may indicate that here to bypass the “Comments to the Author” section, enter your conflict of interest statement in the “Confidential to Editor” section, and submit your "Accept" recommendation.

Reviewer #2: All comments have been addressed

Reviewer #3: All comments have been addressed

**********

2. Is the manuscript technically sound, and do the data support the conclusions?

The manuscript must describe a technically sound piece of scientific research with data that supports the conclusions. Experiments must have been conducted rigorously, with appropriate controls, replication, and sample sizes. The conclusions must be drawn appropriately based on the data presented.

Reviewer #2: Yes

Reviewer #3: Yes

**********

3. Has the statistical analysis been performed appropriately and rigorously?

Reviewer #2: Yes

Reviewer #3: Yes

**********

4. Have the authors made all data underlying the findings in their manuscript fully available?

The PLOS Data policy requires authors to make all data underlying the findings described in their manuscript fully available without restriction, with rare exception (please refer to the Data Availability Statement in the manuscript PDF file). The data should be provided as part of the manuscript or its supporting information, or deposited to a public repository. For example, in addition to summary statistics, the data points behind means, medians and variance measures should be available. If there are restrictions on publicly sharing data—e.g. participant privacy or use of data from a third party—those must be specified.

Reviewer #2: Yes

Reviewer #3: Yes

**********

5. Is the manuscript presented in an intelligible fashion and written in standard English?

PLOS ONE does not copyedit accepted manuscripts, so the language in submitted articles must be clear, correct, and unambiguous. Any typographical or grammatical errors should be corrected at revision, so please note any specific errors here.

Reviewer #2: Yes

Reviewer #3: Yes

**********

6. Review Comments to the Author

Please use the space provided to explain your answers to the questions above. You may also include additional comments for the author, including concerns about dual publication, research ethics, or publication ethics. (Please upload your review as an attachment if it exceeds 20,000 characters)

Reviewer #2: Thank you for your response. All comments have been addressed, resulting in a significantly improved revised manuscript.

Reviewer #3: The manuscript addresses an important and clinically relevant topic and has been substantially improved after revision. The study is generally well conducted and provides valuable findings in the field of radiomics and NSCLC.

Minor Comments:

Careful English language editing is recommended to correct remaining grammatical and typographical errors.

The subgroup findings should be interpreted more cautiously as exploratory observations.

Methodological explanations and figure legends could be slightly clearer for better readability.

Overall, the manuscript is scientifically valuable. Once these minor corrections are addressed, the manuscript may be considered suitable for publication.

**********

7. PLOS authors have the option to publish the peer review history of their article (what does this mean?). If published, this will include your full peer review and any attached files.

If you choose “no”, your identity will remain anonymous but your review may still be made public.

**Do you want your identity to be public for this peer review?**  For information about this choice, including consent withdrawal, please see our Privacy Policy.

Reviewer #2: No

Reviewer #3: No

**********

[NOTE: If reviewer comments were submitted as an attachment file, they will be attached to this email and accessible via the submission site. Please log into your account, locate the manuscript record, and check for the action link "View Attachments". If this link does not appear, there are no attachment files.]

To ensure your figures meet our technical requirements, please review our figure guidelines: https://journals.plos.org/plosone/s/figures

You may also use PLOS’s free figure tool, NAAS, to help you prepare publication quality figures: https://journals.plos.org/plosone/s/figures#loc-tools-for-figure-preparation.

NAAS will assess whether your figures meet our technical requirements by comparing each figure against our figure specifications.

Attachment Submitted filename: Reviewer comments.docx

# Author comment: Author response to Decision Letter 2

**DOI:** 10.1371/journal.pone.0353257.r005

27 May 2026

Response to Reviewer Comments

Response to the review of the paper entitled: “Predicting T790M mutation status in non-small cell lung cancer based on radiomics: a systematic review and meta-analysis”

May 27, 2026

Hongyang Chen

We acknowledge and sincerely appreciate the review. We have revised the manuscript accordingly. In this document, we provide our responses. All the changes are highlighted in red color in the revised manuscript.

Reviewer(s)’ Comments to Author:

1. Careful English language editing is recommended to correct remaining grammatical and typographical errors.

Response: Thank you for your valuable commentary. The manuscript has undergone careful English language editing. All grammatical and typographical errors have been corrected, and the clarity of the text has been improved throughout. The changes are highlighted in red color.

2.The subgroup findings should be interpreted more cautiously as exploratory observations.

Response: Thank you for your helpful feedback. We have already emphasized the exploratory nature of subgroup analyses in the “Limitations” section. Please see page 31, lines 595-603, and all the changes are highlighted in red color.

3.Methodological explanations and figure legends could be slightly clearer for better readability.

Response: Thanks for your considerate suggestion. We have carefully revised the methodology and legends to improve readability. Please see Page 8, lines 183-185�Page 17, lines 312-315, and Page 21, line 371.

Attachment Submitted filename: Response_to_Reviewers_auresp_2.docx

# Editor report: Decision Letter 2

**DOI:** 10.1371/journal.pone.0353257.r006

**Authors:** Hamidreza Montazeri Aliabadi, Bardia Rodd, Bardia Rodd

22 Jun 2026

Predicting T790M mutation status in non-small cell lung cancer based on radiomics: a systematic review and meta-analysis

PONE-D-25-64207R2

Dear Dr. Chen,

We’re pleased to inform you that your manuscript has been judged scientifically suitable for publication and will be formally accepted for publication once it meets all outstanding technical requirements.

Within one week, you’ll receive an e-mail detailing the required amendments. When these have been addressed, you’ll receive a formal acceptance letter and your manuscript will be scheduled for publication.

An invoice will be generated when your article is formally accepted. Please note, if your institution has a publishing partnership with PLOS and your article meets the relevant criteria, all or part of your publication costs will be covered. Please make sure your user information is up-to-date by logging into Editorial Manager at Editorial Manager® and clicking the ‘Update My Information' link at the top of the page. For questions related to billing, please contact billing support.

If your institution or institutions have a press office, please notify them about your upcoming paper to help maximize its impact. If they’ll be preparing press materials, please inform our press team as soon as possible -- no later than 48 hours after receiving the formal acceptance. Your manuscript will remain under strict press embargo until 2 pm Eastern Time on the date of publication. For more information, please contact onepress@plos.org.

Kind regards,

Bardia Rodd, Ph.D.

Academic Editor

PLOS One

Additional Editor Comments (optional):

Authors modified their manuscript according to minor comments. Congratulations

Reviewers' comments:

# Editor report: Acceptance letter

**DOI:** 10.1371/journal.pone.0353257.r007

**Authors:** Hamidreza Montazeri Aliabadi, Bardia Rodd, Bardia Rodd

PONE-D-25-64207R2

PLOS One

Dear Dr. Chen,

I'm pleased to inform you that your manuscript has been deemed suitable for publication in PLOS One. Congratulations! Your manuscript is now being handed over to our production team.

At this stage, our production department will prepare your paper for publication. This includes ensuring the following:

* All references, tables, and figures are properly cited

* All relevant supporting information is included in the manuscript submission,

* There are no issues that prevent the paper from being properly typeset

You will receive further instructions from the production team, including instructions on how to review your proof when it is ready. Please keep in mind that we are working through a large volume of accepted articles, so please give us a few days to review your paper and let you know the next and final steps.

Lastly, if your institution or institutions have a press office, please let them know about your upcoming paper now to help maximize its impact. If they'll be preparing press materials, please inform our press team within the next 48 hours. Your manuscript will remain under strict press embargo until 2 pm Eastern Time on the date of publication. For more information, please contact onepress@plos.org.

You will receive an invoice from PLOS for your publication fee after your manuscript has reached the completed accept phase. If you receive an email requesting payment before acceptance or for any other service, this may be a phishing scheme. Learn how to identify phishing emails and protect your accounts at https://explore.plos.org/phishing.

If we can help with anything else, please email us at customercare@plos.org.

Thank you for submitting your work to PLOS ONE and supporting open access.

Kind regards,

PLOS ONE Editorial Office Staff

on behalf of

Dr. Bardia Rodd

Academic Editor

PLOS One
